# Sphingolipids in Emotional Well‐Being

**DOI:** 10.1111/jnc.70379

**Published:** 2026-02-17

**Authors:** L. S. Kalinichenko, I. Zoicas, C. Mühle, J. Kornhuber, C. P. Müller

**Affiliations:** ^1^ Department of Psychiatry and Psychotherapy, University Clinic Friedrich‐Alexander‐University of Erlangen‐Nuremberg Erlangen Germany; ^2^ Institute of Psychopharmacology, Central Institute of Mental Health University of Heidelberg Heidelberg Germany

**Keywords:** drug consumption, emotional well‐being, emotions, sleep, social interactions, sphingolipids

## Abstract

Emotional well‐being is a multifactorial concept, which comprises not only life quality of human individuals, but also their mental and physical health. It encompasses several key parameters, many of which have behavioral representation in daily life. These include finding positive meaning of life events, ability to maintain supportive and caring social interactions, reward‐oriented behavior, and many others. It is well‐known that the behavioral phenotype is tightly bound to certain physiological and metabolic factors, among which sphingolipid (SL) balance of the organism and especially central nervous system might play an important role. Recent research proposes that SLs mediate multiple components of emotional well‐being. The most abundant brain SL types, ceramides and gangliosides, dynamically shape the composition of protein carrying cellular membranes and overall neuronal plasticity. Multiple studies show the contribution of SLs to normal brain functioning and corresponding beneficial behavioral phenotypes, such as stress resilience, cognitive performance, and social interactions, which determine emotional well‐being. On the other hand, an imbalance in SL metabolism affects normal functioning of cells and thus contributes to the development of several psychiatric disorders, such as depression, anxiety, cognitive decline, schizophrenia, and others. SLs are suggested as a potentially new mechanism of the key behavioral manifestations of emotional well‐being, which might be further investigated as new biomarkers of life quality as well as physical and mental resilience.

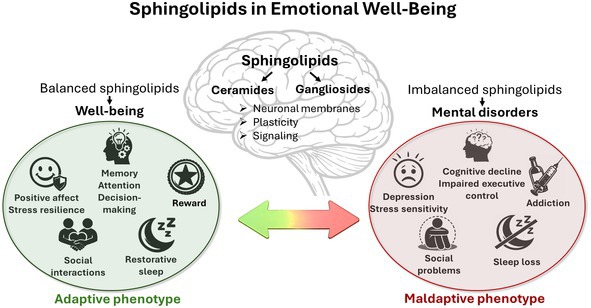

## Graphical abstract

Sphingolipids are essential constituents of neuronal membranes and are increasingly recognized as contributors to the key behavioral manifestations associated with emotional well‐being.

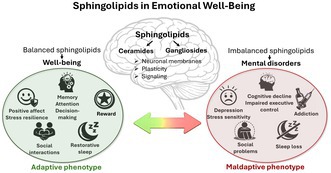

**Abbreviations**
5‐HIAA: 5‐hydroxyindoleacetic acid
5‐HT: serotonin
AC: acid ceramidase
ACh: acetylcholine
ADHD: attention deficit hyperactivity disorder
AMPA: α‐amino‐3‐hydroxy‐5‐methyl‐4‐isoxazolepropionic acid
AMPK: AMP‐activated protein kinase
ASD: autism spectrum disorder
ASM: acid sphingomyelinase
ASMtg: mouse line with whole‐body ASM overexpression
ASMtg^fb^
: mouse line with forebrain‐specific ASM overexpression
AUC‐ROC: area under the receiver operating characteristic curve
AUD: alcohol use disorder
BDNF: brain‐derived neurotrophic factor
BMI: body mass index
BSID‐II: Behavior Rating Scale of the Bayley Scales of Infant Development
CAPP: ceramide‐activated protein phosphatase
CerS: ceramide synthases
CerS6KO: mice with inactive ceramide synthase 6 gene
CERT: ceramide transfer protein
CPP: conditioned place preference
CSF: cerebrospinal fluid
CUS: chronic unpredictable stress
DA: dopamine
DBS: dried blood spots
DH: dorsal hippocampus
dhCer: dihydroceramides
dhSM: dihydrosphingomyelin
DS: dorsal striatum
DSM: diagnostic and statistical manual of mental disorders
FC: frontal cortex
fEPSP: field excitatory postsynaptic potentials
FIASMA: functional inhibitors of ASM activity
Fro: genetically modified mice with NSM hypoexpression
GalCer: galactosylceramide
GBA: glucocerebrosidase
GluCer: glucosylceramide
GPCR: G‐protein‐coupled receptor
HAB: rats selectively bred for high anxiety behavior
HADS: Hospital Anxiety and Depression Scale
HAM‐D: Hamilton Depression Scale
HexCer: hexosylceramide
IPSCs: induced pluripotent stem cells
iRBD: idiopathic rapid eye movement sleep behavior disorder
LAB: rats selectively bred for low anxiety behavior
LacCer: lactosylceramide
L‐PDMP: L‐threo‐1‐phenyl‐2‐decanoylamino‐3‐morpholino‐1‐propanol
LPS: lipopolysaccharide
MAPK: mitogen‐activated protein kinase
MDD: major depressive disorder
NA: noradrenalin
Nac: nucleus accumbens
NC: neutral ceramidase
NGF: nerve growth factor
NMDA‐R: N‐methyl‐D‐aspartate receptor
NSC: neural stem cell
NSM: neutral sphingomyelinase
p75NTR: p75 neurotrophin receptor
PFC: prefrontal cortex
PKCδ: protein kinase C delta
PKCε: protein kinase C epsilon
PLD: phospholipase D
PSD: post‐synaptic density
PTSD: posttraumatic stress disorder
ROS: reactive oxygen species
S1P: sphingosine‐1‐phosphate
SFC: social fear conditioning
SL: sphingolipid
SM: sphingomyelin
Sph: sphingosine
SPT: serine palmitoyl transferase
ST: sialyltransferase
TLR: toll‐like receptor
TNFα: tumor necrosis factor α
TrkB: tyrosine kinase receptor B
VH: ventral hippocampus
VS: ventral striatum
wt: wild type

## 1 Introduction

The World Health Organization defines health as a state of full mental, physical and social well‐being, rather than just the absence of disease. Well‐being is a multi‐dimensional phenotype indicating how positive an individual feels generally and about life overall. The National Institutes of Health describe well‐being, and particularly emotional well‐being, as “… an overall positive state of one's emotions, life satisfaction, sense of meaning and purpose, and ability to pursue self‐defined goals” National Institutes of Health. Emotional Well‐Being: High Priority Research Networks (U24, Clinical Trial Optional 2018; Park et al. 2023). Well‐being includes the absence of mental health problems and the presence of self‐acceptance (positive self‐evaluation), personal growth, belief that a person has a purposeful and meaningful life, positive relations with others, environmental mastery (an ability to manage life and environment), personal growth, and autonomy (a sense of determination) (Ryff and Keyes 1995; Ryff 1989).

Voluminous research speaks about the importance of mental health for overall well‐being; it is not only desirable but may causally contribute to healthy aging and longevity (Ngamaba et al. 2017; Kushlev et al. 2020). Certain components of emotional well‐being, such as high levels of life satisfaction or sense of purpose, predict physical health (Chida and Steptoe 2008; Pressman et al. 2019; Zaninotto and Steptoe 2019). Whereas poor well‐being, which could be manifested in anxiety, depression, addiction, and lack of social interactions, can increase the risk for physical diseases, a positive interaction between emotional well‐being and physical health was widely shown (Park et al. 2023).

Emotional well‐being is considered as an immediate and direct “reward” from the behavioral representations for a lifestyle. Although behavioral patterns associated with lifestyle occur in the context of culture, life circumstances, resources, and life course, there are common types of behavior with a strong effect on emotional well‐being. Emotional well‐being includes affective and cognitive well‐being, emotional balance, healthy eudemonic and hedonic behavior, social acceptance and relationships (Park et al. 2023). Some of these phenotypes could be considered as a key mechanism of well‐being, which are mildly affected by life context and social environment. It is, for example, finding positive meaning in ordinary events and within life adversity as a marker of stress resilience and response to life problems. Finding positive meaning predicts high degree of well‐being and health (Davis et al. 1998) and requires high level of attention and cognitive flexibility (Fredrickson and Joiner 2002; Park et al. 2023). Ability to create and maintain supportive social interactions and caring community relationships is also considered as protective factors against psychopathology over the life course (Bluth et al. 2017). Moreover, other behavioral representations for a lifestyle, such as sleep quality, healthy eating or physical activity mediate overall life satisfaction and well‐being (Berkowitz et al. 2021). Altogether, psychological aspects of well‐being are increasingly recognized as fundamental components of healthy human functioning.

Although well‐being is predominantly considered by psychologists as a subjective construct, accumulating evidence indicates that it constitutes an objective and measurable neurobiological phenomenon. Well‐being is not merely a mental state but also a bodily state, encompassing the brain (Brandt et al. 2025). Findings from behavioral and molecular genetic studies further demonstrate a substantial contribution of biological and physiological factors to inter‐individual differences in well‐being. Twin studies have estimated the heritability of well‐being to be approximately 40% (Vries et al. 2022; Nes and Røysamb 2015; Van de Weijer et al. 2020; Bartels 2015). More recently, genome‐wide association studies have linked specific genetic variants to well‐being (Baselmans et al. 2019; Okbay et al. 2016; Turley et al. 2018), with evidence for preferential enrichment of genes differentially expressed in the subiculum and enrichment for GABAergic interneurons (Baselmans et al. 2019). Collectively, these genetic findings offer important insights and provide a foundation for elucidating the physiological mechanisms underlying well‐being (Brandt et al. 2025). Although well‐being is being considered as mainly a psychological concept, it is strongly based on innate and acquired biological mechanisms.

Emerging research has also linked well‐being with many physiological factors, such as gut microbiota, inflammatory processes, cortisol and other hormones, as well as neurotransmitters such as serotonin (Lee, Yoon, et al. 2020; Vries et al. 2022; Ryff et al. 2004). A recent study of Berkowitz et al. (2021) performed on a large national survey of middle‐aged American adults for the first time showed blood sphingolipids (SLs) as principally new markers of well‐being. Total serum ceramide levels were inversely linked with environmental mastery (an individual's ability to effectively manage their environment and make use of its resources to achieve their goals), purpose in life, and self‐acceptance. Detailed analysis of various SL species emphasized these interactions. Particularly, levels of dihydroceramides dhCer24 and dhCer24:1, hexosylceramide HexCer20, lactosylceramide LacCer18:1, and sphingomyelin SM20:1 were significantly associated with environmental mastery. Six species of ceramides (Cer16, Cer18, Cer20, Cer22, Cer24, and Cer24:1) negatively correlated with purpose in life and self‐acceptance, while only three of them (Cer18, Cer20, and Cer22) were significantly associated with purpose in life after covariate adjustments. The strong negative association between ceramides and environmental mastery was partly mediated by health behaviors, specifically by body mass index (BMI) and sleep quality (Berkowitz et al. 2021). In line, a genome‐wide association study showed the association between the two single nucleotide polymorphisms, rs2574985 and rs2099527, of the *SGMS1* gene, which codes for a crucial enzyme of the SL system, sphingomyelin synthase, and subjective well‐being or life satisfaction (Okbay et al. 2016). Altogether, SLs are being currently proposed as principally new biomarkers of emotional well‐being and mental health.

Although the direct interactions between SLs and emotional well‐being are poorly studied yet, recent studies widely consider the role of these lipid molecules in various aspects of mental health, which could contribute to well‐being. In this review we will discuss the contribution of two big groups of SLs, ceramides and gangliosides, as the key SLs of biological membranes and particularly of lipid rafts, which determine the key role of SLs in normal and pathological functioning of the organism. Mental well‐being exists as a complex continuum, with states of happiness and well‐being at one end and mental health crises, such as anxiety and depression, social behavior disorders, at the other (Bluth et al. 2017). Therefore, we will also consider the role of these SLs in the mechanisms contributing to deviations from well‐being, particularly mental disorders. Many aspects of the current emotional well‐being definition refer to concepts based on human self‐reports and are not attainable in non‐human species (Park et al. 2023). However, there are certain biological concepts determining well‐being, which can be tested experimentally in rodents. These include the emotional phenotype, the response to stress and stress resilience, cognitive performance and flexibility, executive functions, social interactions, the ability to maintain healthy behavior including sleep quality (Kahneman et al. 1999).

## 2 The Sphingolipid System

SLs represent one of the major classes of lipids ubiquitously present in all eukaryotic cells. Alongside cholesterol and glycerophospholipids, SLs constitute the most prevalent lipids of biological membranes (Holthuis et al. 2001). This lipid group consists of ceramides, sphingomyelins (SMs), and glycosphingolipids, including gangliosides, cerebrosides, and sulfatides (Fahy et al. 2005). SLs play a fundamental role in maintenance of the functional integrity of the nervous system (Olsen and Færgeman 2017; Piccinini et al. 2010; Echten‐Deckert and Alam 2018). SLs and particularly ceramides participate in numerous essential physiological processes, such as programmed cell death and differentiation, cell proliferation, cell cycle arrest, cellular migration, senescence, necrosis, necroptosis, autophagy, mitophagy, cytoskeletal rearrangement, cell‐to‐cell recognition, adhesion, and other functions (Hannun and Obeid 2011). Gangliosides constitute the predominant class of lipids in brain gray matter and neurons, whereas galactosylceramides (GalCer) and sulfatides are particularly enriched in oligodendrocytes and the myelin sheath (Aureli et al. 2016; Kuznetsov et al. 2011; Posse de Chaves and Sipione 2010; Olsen and Færgeman 2017). Due to their abundance in the central nervous system, SLs significantly influence a range of neuropsychiatric and neurological conditions. In particular, disturbances in the SL rheostat have been implicated as critical pathogenic mechanisms underlying major depressive disorder (MDD), bipolar disorder, schizophrenia, substance use disorder, amyotrophic lateral sclerosis, and cerebral ischemic injury (Chestnykh et al. 2025; Blasco et al. 2017; Brodowicz et al. 2018; Gulbins et al. 2018; Gulbins et al. 2013; Hillard 2005; Zoicas, Huber, et al. 2020; Kalinichenko, Abdel‐Hafiz, et al. 2021; Kalinichenko et al. 2025; Müller et al. 2017; Wenk 2005; Schneider et al. 2017).

### 2.1 Structure and Metabolism of Sphingolipids

The SL family comprises over 300 distinct molecular species, all sharing a common sphingosine (Sph) backbone (Hannun and Obeid 2018). The conjugation of a fatty acid to the Sph base results in the formation of ceramides, while further attachment of choline or ethanolamine head groups is required for the biosynthesis of SMs. Ceramides and SMs may contain up to 30 carbon atoms with various degrees of saturation; for example, Cer18:0 denotes a saturated chain, while Cer18:1 indicates a desaturated chain (Fahy et al. 2005). It should be emphasized that multiple studies indicate different roles of specific SL species in physiological and pathological states (Kornhuber et al. 2013; Kalinichenko, Abdel‐Hafiz, et al. 2021; Chestnykh et al. 2025; Müller et al. 2017). Therefore, in our review we focus on various species rather than total levels of SLs. However, despite the clear biochemical classification and nomenclature of SL species, many researchers tend to simplify the naming of single species. For example, some researchers refer to Cer24, which might correspond to Cer24:0, Cer24:1, or even a mixture of both species. As it is not always possible to determine the exact species investigated, we are using the original terminology used by the authors in the cited studies.

Ceramides, as the key metabolic hub within SL metabolism, can be synthesized by three main pathways: the de novo pathway, the hydrolysis of complex sphingolipids, and the salvage pathway. The de novo pathway is a reaction of serine and palmitoyl‐coenzyme resulting in the generation of dihydroceramide followed by a transformation to ceramide. This pathway is catalyzed by serine palmitoyl transferase (SPT), sphingosine‐1‐phosphatase, six isoforms of ceramide synthases (CerS), and dihydroceramide desaturase (Hannun and Obeid 2011; Trayssac et al. 2018; Hannun and Obeid 2018).

The sphingomyelinase pathway of ceramide synthesis involves the breakdown of SM into ceramide catalyzed by sphingomyelinases. There are three main families of sphingomyelinases: acid sphingomyelinases (ASM), neutral sphingomyelinases (NSM), and alkaline sphingomyelinases, which differ in their optimal pH conditions and intracellular localisations (Trayssac et al. 2018; Hannun and Obeid 2011). Among the sphingomyelinase enzymes, acid sphingomyelinase (ASM) and neutral sphingomyelinase (NSM) are the best characterized and shown to mediate several biological processes and disorders (Duarte et al. 2020). ASM activity has been detected in a variety of mammalian extracellular fluids, including serum, cerebrospinal fluid, urine, saliva, tears, and synovial fluid (Takahashi et al. 2000) as well as in several peripheral organs and all regions of the brain (Spence et al. 1979; Treleaven et al. 2011). Several isoforms of NSM, coded by the genes *SMPD2*, *SMPD3*, *SMPD4*, and *SMPD5*, have been identified in various cell compartments (Clarke et al. 2011; Deevska and Nikolova‐Karakashian 2017). NSM is particularly enriched in neural tissues (Horres and Hannun 2012; Spence et al. 1979). The enzymes catalyzing degradation of ceramide back to SM are the ceramidases. To date, five human ceramidases have been identified: acid ceramidase (AC), neutral ceramidase (NC), and three alkaline ceramidases (Duarte et al. 2020).

In the salvage pathway of ceramide synthesis, a bioactive signaling lipid named sphingosine‐1‐phosphate (S1P) is converted into Sph and then to ceramide through enzymatic reactions catalyzed by S1P phosphatase and CerS (Hannun and Obeid 2011). This recycling pathway plays a critical role in the regulation of programmed cell death, as the balance between the anti‐apoptotic S1P and the pro‐apoptotic ceramide determines apoptosis and autophagy within the cell (Maceyka et al. 2002). S1P is one of the key signaling lipids with its own G‐protein‐coupled receptors, which affects multiple physiological processes (Proia and Hla 2015). However, in this review we do not focus on this bioactive molecule due to its specific role in cell signaling.

Complex SLs including gangliosides and cerebrosides contain sugar residues, particularly complex carbohydrates, added to the ceramide core. Gangliosides constitute a large and structurally diverse family, distinguished by variations in ceramide composition, glycosidic linkage positions, sugar stereochemistry, and the number of neutral sugar units and sialic acid residues within their structure. According to the number of sialic acid moieties, gangliosides are classified into GM (mono‐sialylated), GD (di‐sialylated), GT (tri‐sialylated), and GQ (quadra‐sialylated) subgroups. The number in the name of gangliosides correspond to the migration order during thin layer chromatography (e.g., GM3 > GM2 > GM1). More complex gangliosides belonging to the a‐, b‐, and c‐series are derived from GM3, GD3, and GT3, respectively (Palmano et al. 2015; Sugiura et al. 2008; Yu et al. 2009, 2011).

### 2.2 Biological Functions of Sphingolipids

Recent studies have demonstrated that, beyond their classical roles in structural integrity, SLs directly and indirectly modulate cell signaling. Ceramides, for instance, may function as second messengers by binding to ceramide‐activated protein phosphatases (CAPPs) as well as other intracellular targets including protein kinase C zeta (Müller et al. 1995; Blitterswijk et al. 2003). The diverse effects of ceramides on cellular signaling underlie their involvement in numerous essential processes, such as cell survival (Westwick et al. 1995), proliferation (Olivera et al. 1997), differentiation (Okazaki et al. 1989), growth arrest (Bourbon et al. 2002), apoptosis (Obeid et al. 1993), and the modulation of membrane permeability (Siskind et al. 2002).

Another fundamental function of SLs is their contribution to signaling processes in biological membranes. In neurons, membrane domains with lower fluidity enriched in lipids, commonly referred to as “lipid rafts,” are highly prevalent within the lipid bilayer (Fujii et al. 2002; George and Wu 2012). These domains are being formed due to biochemical interactions between SLs and cholesterol (Gerkin et al. 2007) and are able to rapidly migrate within lipid‐disordered membrane regions (Varma and Mayor 1998; Gaus et al. 2003; Van Blerkom and Zimmermann 2016). The biological significance of lipid domains is related to their enrichment in functional proteins, particularly G‐protein‐coupled receptors (Kornhuber et al. 2014; Schneider et al. 2017). Alterations in the composition of these lipid domains may affect receptor affinity and internalization and determine signaling (Nothdurfter et al. 2013, 2010). Moreover, the lipid domains largely determine the protein composition of the PSD and are pivotal for the signaling of PSD‐associated receptors, such as subunits of the N‐methyl‐D‐aspartate receptor (NMDA‐R) (Suzuki 2002; Hering et al. 2003; Swanwick et al. 2009). This way, the architecture and distribution of lipid domains exert profound effects on cell signaling, synaptic transmission, and neuronal plasticity (Kalinichenko et al. 2024; Pfeiffer et al. 2001; Stancevic and Kolesnick 2010).

Alterations in the composition of lipid domains occurring upon various stimuli considerably affect the physical properties of the plasma membrane as well as its plasticity (Grecksch et al. 1991; Greene et al. 2008; Grimm et al. 2017; Guirland and Zheng 2007; Gulbins et al. 2018). Several mechanisms exist for modulating the lipid composition of ceramide‐enriched domains. One of them is based on the activity of lipid‐metabolizing enzymes localized within the lipid domains (He et al. 2010; Guirland and Zheng 2007). For instance, inhibition of NSM significantly reduces the amount of annexin 6, a lipid raft protein marker at synaptic membranes (Heneka et al. 2015). This is associated with the blockage of phosphorylation and clustering of the NR1 subunit of NMDA, NMDA‐triggered Ca^2+^ influx, and excitatory postsynaptic currents in cultured hippocampal neurons (Wheeler et al. 2009). Furthermore, NSM inhibition also alters the expression of multiple NMDA receptor and α‐amino‐3‐hydroxy‐5‐methyl‐4‐isoxazolepropionic acid (AMPA) receptor subunits (Tabatadze et al. 2010; Kalinichenko, Abdel‐Hafiz, et al. 2021). As another example, ASM activation by lipopolysaccharide (LPS) leads to ceramide generation within lipid domains, followed by PKC‐ζ phosphorylation, the assembly of Toll‐like receptor 4 (TLR4), activation of the mitogen‐activated protein kinase (MAPK) pathway, and TNFα release (Rozenova et al. 2010; Płóciennikowska et al. 2015). In summary, the sphingomyelin/ceramide rheostat controlled by the metabolizing enzymes plays essential roles in lipid domain formation, downstream cellular signaling, and cell plasticity.

Altogether, involvement of various SLs in essential mechanisms of cellular function, their high abundance within brain tissue, and effects on cell signaling underscore the crucial role of these lipids in physiological processes in the brain. These emerging data support the hypothesis that SLs represent an independent pathway determining several neurobiological processes and therefore the contribution of SLs to processes determining emotional behavior under physiological and pathological conditions. In this review we discuss two key members of the SL family, ceramides and gangliosides, as main compounds determining emotional well‐being in mammals.

## 3 Sphingolipids and Components of Emotional Well‐Being

### 3.1 Sphingolipids in Emotional Regulation

Emotional resilience is the ability to adapt and cope with stressful situations and adversity. It is a dynamic process characterized by interaction with biological, psychological, and social systems as a response to a stressful event, collectively contributing to favorable health outcomes. Resilience is positively correlated with emotional well‐being and negatively correlated with poor mental health (Herrman et al. 2011; Klainin‐Yobas et al. 2021). Among other markers, diminished expression of alkaline ceramidase 2, a catabolic ceramide enzyme, was described in the medial prefrontal cortex (mPFC) of stress susceptible compared to resilient mice in a social defeat model (Yang, Sun, et al. 2020). These data indicate that the SL system might, to a certain extent, determine stress resilience and associated emotional well‐being.

Two studies also showed pronounced changes in brain lipidome after stress exposure (Oliveira et al. 2016; Miranda et al. 2019) in a chronic unpredictable stress (CUS) model. CUS exerted strong effects on the SL composition of rat brain in a region‐specific manner. In the PFC, CUS induced a massive increase in the levels of ceramides Cer16:0, Cer16:1, Cer18:1, Cer22:1, and Cer26:1, as well as lactosylceramides LacCer 18:0, LacCer24:0, and LacCer26:1, while levels of other species remained unchanged. In line, the levels of most studied SMs (SM16:0, SM20:0, SM22:0, SM24:0, SM26:0) and dihydroSMs (dhSM16:0, dhSM16:1, dhSM18:0, dhSM18:1, dhSM20:0, dhSM22:0, dhSM22:1, dhSM24:0, dhSM24:1, dhSM26:0, dhSM26:1, dhSM26:2 with only exception for dhSM20:1; Table 1) increased in the PFC of male rats exposed to CUS. Blood corticosterone concentration negatively correlated with the total SM level in the PFC (Oliveira et al. 2016). A similar increase in the total amount of ceramides and a decrease in SM concentration were observed in the hippocampus, but not in the amygdala or cerebellum (Oliveira et al. 2016), suggesting a brain region‐specific involvement of SLs in stress and possibly resilience. Miranda et al. (2019) investigated the effects of chronic corticosterone exposure along the hippocampal longitudinal axis and observed that changes in the ceramide levels were significantly more pronounced in the ventral hippocampus (VH). While the concentrations of Cer20:0, Cer20:1, Cer24:1, Cer26:0, and Cer26:1 were enhanced in the VH of corticosterone‐treated male rats, only an increase in Cer22:1 was observed in the dorsal hippocampus (DH). On the contrary, levels of HexCer16:0, HexCer18:0, HexCer18:1, HexCer20:0, HexCer26:0, HexCer26:1, and LacCer20:0 and LacCer26:1 were increased in the DH, but not in the VH. The concentrations of SMs and dhSMs were intact in the DH, but an increase in the levels of dihydrosphingomyelins dhSM16:1, dhSM20:0, dhSM20:1, dhSM22:1, dhSM26:0, dhSM26:1, and dhSM26:2 were observed in the VH (Miranda et al. 2019). In this study, mRNA levels of enzymes of the sphingomyelinase pathway, *Smpd1* coding for ASM and *Smpd2* coding for NSM, remained unaltered in the PFC of stressed rats (Oliveira et al. 2016). This is in line with the study of Gulbins et al. (2013) showing no effects of corticosterone on ASM activity or expression in the hippocampus of mice. However, no changes in the total ceramide level were found (Gulbins et al. 2013). Chronic neurogenic stress was also shown to increase total ceramide level and to reduce SM concentration in the hippocampus, but not in the neocortex, liver or blood serum of rats for at least 8 days after stress exposure (Babenko et al. 2016). Stress exposure was associated with an increase in the expression of the AC gene *ASAH1* (Lucki and Sewer 2008; Urs et al. 2006).

**TABLE 1:** Sphingolipids in emotional regulation: Changes in tissue levels of ceramides and sphingomyelins in preclinical and clinical studies.

Preclinical studies				
Type of stress	Species	Changes	Tissue	References
Chronic unpredictable stress	Male rats	↑ Cer16:0, Cer16:1, Cer18:1, Cer22:1, and Cer26:1 ↑ LacCer 18:0, LacCer24:0, and LacCer26:1 ↑ SM16:0, SM20:0, SM22:0, SM24:0, SM26:0 ↑ dhSM16:0, dhSM16:1, dhSM18:0; dhSM18:1, dhSM20:0, dhSM22:0, dhSM22:1, dhSM24:0, dhSM24:1, dhSM26:0, dhSM26:1, dhSM26:2	Prefrontal cortex	Oliveira et al. 2016
	Male rats	↑ total Cer and HexCer ↓ total SM	Hippocampus	Xue et al. 2020
	Male rats	↑ Cer18:0/23:0, Cer18:1/24:0, SM18:1/18:0, SM18:2/18:0, SM18:0/18:0, SM18:1/24:1, SM18:1/25:0, SM18:2/24:1 ↓ dhSPh1‐5, Cer18:0/16:0, Cer16:0/20:0	Hippocampus	Gong et al. 2024
Chronic corticosterone exposure	Male rats	↑ Cer20:0, Cer20:1, Cer24:1, Cer26:0, and Cer26:1 ↑ dhSM16:1, dhSM20:0, dhSM20:1, dhSM22:1, dhSM26:0, dhSM26:1, and dhSM26:2	Ventral hippocampus	Miranda et al. 2019
		↑ Cer22:1 ↑ HexCer16:0, HexCer18:0, HexCer18:1, HexCer20:0, HexCer26:0, HexCer26:1 and LacCer20:0 and LacCer26:1	Dorsal hippocampus	
Single prolonged stress	Male mice	↑ Cer(d18:0+pO/24:0+O)+HCOO ↑ hexCer HexCer(d18:0/24:0+O)+H ↑ HexCer(d18:1/18:1)+H, HexCer(d18:1/22:1)+H, HexCer(d18:1/24:1)+H, HexCer(d18:1/25:0)+H, HexCer(d58:4)+H ↑ SM(d22:1/16:0)+HCOO, SM(d34:1)+H, and SM(d36:0)+H	Hippocampus	Zhou et al. 2022
		↑ Cer(d20:1)+H	Prefrontal cortex	
Chronic neurogenic stress	Rats (no sex reported)	↑ total Cer ↓ total SM	Hippocampus	Babenko et al. 2016
Chronic psychosocial stress	Male mice	↑ Cer16:0 ↓ Cer22:0 and Cer24:0	Liver	Reichel et al. 2018

Clinical studies				
Disorder	Gender	Changes	Tissue	References
Posttraumatic stress disorder	Males	↓ Cer d18:1/24:0, Cer d18:0/24:0 ↓ SM d18:1/22:0, SM d18:0/24:0, and SM d18:1/24:0	Plasma	Konjevod et al. 2022
	Males	↑ Cer14:0, Cer16:0, Cer18:0, Cer18:1, Cer20:0, Cer20:1, Cer22:0, Cer22:1, Cer24:0, Cer24:1, Cer26:0, Cer26:1 ↑ dhCer16:0, dhCer18:0, dhCer20:0, dhCer22:0, dhCer22:1, dhCer22:2, dhCer24:0, dhCer24:1, dhCer26:0, dhCer26:1 ↑ HexCer14:0, HexCer16:0, HexCer18:0, HexCer18:1, HexCer20:0, HexCer20:1, HexCer22:0, HexCer22:1, HexCer24:0, HexCer24:1, HexCer26:0, HexCer26:1 ↑ LacCer14:0, LacCer16:0, LacCer18:0, LacCer18:1, LacCer20:0, LacCer20:1, LacCer22:0, LacCer22:1, LacCer24:0, LacCer24:1 ↑ SM14:0, SM16:0, SM18:0, SM18:1, SM20:0, SM20:1, SM22:0, SM22:1, SM24:0, SM24:1, SM26:0, SM26:1	Plasma	Kuan et al. 2022
	No gender reported	↑ Cer18:0	Plasma	Hammad et al. 2012
	Females	↑ Cer22:6	Plasma	Bhargava et al. 2024
	Males	↑ Cer d36:1, Cer d34:1, Cer d38:1, Cer d31:1, Cer d42:0, Cer d18:1/23:0, Cer d39:1, Cer d40:0, Cer d42:1, Cer d40:1, Cer d42:1, Cer d42:2, Cer d42:2) and SMs (SM d36:2, SM d34:2, SM d36:1, SM d41:1, SM d36:0, SM d34:1, SM d41:2, SM d40:1, SM d33:1, SM d40:0, SM d42:1, SM d38:2, SM d39:1, SM d38:0, SM d37:1, SM d34:0, SM d42:1, SM d38:1	Plasma	Bhargava et al. 2024
Major depressive disorder	No gender reported	↑ Cer16:0, Cer18:0, Cer20:0, Cer24:1, and Cer26:1	Plasma	Gracia‐Garcia et al. 2011
	Male and female	↓ total SM ↓ SM26:1, SM39:1 and SM39:2	Plasma	Liu et al. 2016; Moaddel et al. 2018
Major depressive disorder associated with bipolar disorder	Male and female	↑ Cer16:0, Cer18:0, Cer20:0, Cer22:0, Cer24:0 and Cer24:1	Plasma	Brunkhorst‐Kanaan et al. 2019

Abbreviations: Cer, ceramide; dhCer, dihydroceramide; dhSM, dihydrosphingomyelin; dhSPh, dihydrosphingosine; HexCer, hexosylceramide; LacCer, lactosylceramide; SM, sphingomyelin.

The peripheral SL system also responds to stress. Chronic psychosocial stress in male mice was associated with an increase in Cer16:0 level and a decrease in the concentrations of Cer22:0 and Cer24:0 in the liver. mRNA of enzymes of the ceramide metabolism, such as Cers5, Cers6, glucocerebrosidase, glucocerebrosidase 2, Ormdl2, and sphingomyelin phosphodiesterase acid‐like 3B, was significantly higher in the liver of stressed mice. Hepatic ASM activity was also enhanced after stress (Reichel et al. 2018). Altogether, pronounced changes in the SL system are observed after stress and might determine the negative effects of stress exposure.

One of the severe consequences of intense stress can be a posttraumatic stress disorder (PTSD). It is a complex neuropsychiatric disorder, which develops in certain individuals after experiencing or witnessing a traumatic event. It is always stress‐associated and in most of the cases comorbid with negative alterations in mood and thinking (Konjevod et al. 2022). Several clinical studies showed changes in the SL system in patients with PTSD. A study of Konjevod et al. (2022) on male patients with combat PTSD revealed decreased plasma concentration of ceramides Cer d18:1/24:0, Cer d18:0/24:0 and SMs SM d18:1/22:0, SM d18:0/24:0, and SM d18:1/24:0 in PTSD patients compared to healthy controls. On the contrary, levels of SM d18:2/18:0 and SM d18:2/20:0 were increased in the plasma of PTSD patients (Konjevod et al. 2022). In a male cohort of World Trade Center responders who were exposed to the 9/11 attacks in New York City, a massive increase in plasma SLs levels (57 out of 61 measured ceramides, dhCer, lacCer and SMs; Table 1) was shown (Kuan et al. 2022). In a small clinical study by Hammad et al. (2012) performed on 8 patients with PTSD, Cer18:0, but not other long‐chain ceramides, were significantly elevated in the plasma of individuals with PTSD compared to healthy controls. In line, plasma ASM activity, S1P, and dhS1P were higher in PTSD patients. Although the gender of PTSD patients was not reported in this study (Hammad et al. 2012), another study showed pronounced gender differences in PTSD patients. Severe PTSD was associated with 22% and 5% of altered lipid metabolites in men and women, respectively. Total ceramide and SM levels correlated with the PTSD checklist of the DSM‐IV (PCL), a self‐report measure used to assess the severity of chronic PTSD symptoms, in males, but not in females. A high PCL score in females was associated with significant changes in plasma Cer22:6 level. As distinct from females, a high PCL score in male PTSD patients was associated with an increase in practically all studied species of ceramides (Cer d36:1, Cer d34:1, Cer d38:1, Cer d31:1, Cer d42:0, Cer d18:1/23:0, Cer d39:1, Cer d40:0, Cer d42:1, Cer d40:1, Cer d42:1, Cer d42:2, Cer d42:2) and SMs (SM d36:2, SM d34:2, SM d36:1, SM d41:1, SM d36:0, SM d34:1, SM d41:2, SM d40:1, SM d33:1, SM d40:0, SM d42:1, SM d38:2, SM d39:1, SM d38:0, SM d37:1, SM d34:0, SM d42:1, SM d38:1). Therefore, it might be suggested that the SL pathway is crucial for the pathogenesis of PTSD in males, but to a lower extent in females (Bhargava et al. 2024). Similar patterns were observed in male mice in a PTSD model of modified single prolonged stress. A significant increase in several SL species, such as ceramide Cer(d18:0+pO/24:0+O)+HCOO, hexCer HexCer(d18:0/24:0+O)+H, HexCer(d18:1/18:1)+H, HexCer(d18:1/22:1)+H, HexCer(d18:1/24:1)+H, HexCer(d18:1/25:0)+H, HexCer(d58:4)+H, and SMs SM(d22:1/16:0)+HCOO, SM(d34:1)+H, and SM(d36:0)+H, were observed in the hippocampus of the PTSD group. In the PFC of these mice, only the level of Cer(d20:1)+H, but not other SLs, were enhanced (Zhou et al. 2022). In a classical fear conditioning paradigm, a model of specific attributes of PTSD, it was shown that ASM does not affect fear learning as both ASM overexpressing and ASM knockout mice display comparable fear conditioning to wild type (wt) littermates. However, ASM overexpression enhances the expression of contextual fear in both male and female mice, while ASM deficiency specifically reduces the expression of contextual fear in male mice. In contrast, the expression of cued fear is not regulated by ASM as all genotypes displayed similar tone‐elicited freezing levels (Zoicas and Kornhuber 2022).

Another crucial disorder often associated with stress is MDD. Multiple clinical studies emphasize the significance of SLs in the pathogenesis of depression. Patients with MDD were shown to have increased level of Cer16:0, Cer18:0, Cer20:0, Cer24:1, and Cer26:1 compared to individuals with a past depressive episode more than 2 years prior, as well as to healthy controls (Gracia‐Garcia et al. 2011). In line, patients with MDD and bipolar disorder showed increased plasma levels of Cer16:0, Cer18:0, Cer20:0, Cer22:0, Cer24:0 and Cer24:1 compared to controls (Brunkhorst‐Kanaan et al. 2019; Schumacher, Edwards, et al. 2022). Total plasma SM levels are reduced in patients with MDD (Liu et al. 2016; Moaddel et al. 2018). Species analysis showed decreased plasma levels of SM26:1 (Moaddel et al. 2018), SM39:1 and SM39:2 (Liu et al. 2016), but not of SM16:0, SM16:1, SM24:0 and SM24:1 (Moaddel et al. 2018) in patients with MDD. Importantly, total ceramide level correlated with the Hamilton Depression Scale (HAM‐D) score of depression in MDD patients, while plasma levels of Cer16:0 and SM18:1 correlated with Hospital Anxiety and Depression Scale (HADS) depression subscale score in patients with coronary artery disease (Dinoff et al. 2017; Schumacher, Edwards, et al. 2022). A study by Liu et al. (2016) showed significant correlations between HAM‐D scores and plasma levels of SM36:1, SM36:2, SM38:1, SM38:2, SM40:3, SM39:1, SM41:1, and SM42:3 in patients with MDD (Liu et al. 2016). Moreover, the SM23:1/SM16:0 ratio was negatively correlated with the severity of depressive symptoms measured by HADS‐A and HADS‐D scores (Demirkan et al. 2013). Treatment with valproate, one of the most widely used drugs for the treatment of bipolar disorder, is associated with an increase in dhCer16, dhCer18, and dhCer20 in yeast cell cultures (Jadhav et al. 2016).

Preclinical studies also emphasize the role of the SL system in depression‐ and anxiety‐like behavior. Exposure of male mice to CUS induced an increase in the total level of ceramide and hexosylceramide (HexCer), but decreased the amount of SM in the hippocampus (Xue et al. 2020). In line, CUS‐induced increase in total ceramide levels was observed in the PFC, hippocampus, and nucleus accumbens (Nac) as well as enhanced ASM activity in the PFC and hippocampus of male mice (Chen et al. 2022). The severity of the depression‐like behavior positively correlated with ceramide amount and negatively correlated with SM level in the hippocampus. Repetitive transcranial magnetic stimulation normalized the levels of these SLs (Xue et al. 2020). In another study, CUS induced a decrease in the relative amount of dhSPh1‐5, Cer18:0/16:0, Cer16:0/20:0, but enhanced levels of Cer18:0/23:0, Cer18:1/24:0, SM18:1/18:0, SM18:2/18:0, SM18:0/18:0, SM18:1/24:1, SM18:1/25:0, SM18:2/24:1 in the hippocampus of male rats. The expression of NSM and sphingosine kinase was reduced, while the levels of CerS2 and SPT were enhanced in stressed rats (Gong et al. 2024). CUS was shown to increase total levels of ceramides, but reduce concentrations of ceramide‐1‐phosphate, SM, SM‐phytosphingosine, and gangliosides GM1, GM3, GM2, and GD3 in the DH of male mice. A detailed analysis showed stress‐induced changes in the concentrations of the following SL species: SM d14:0/24:1, SM d36:1, SM d34:0, SM d36:2, SM d18:1/18:4, SM d36:6, SM d34:2, SM d42:2, SM d42:1, SM d18:2/21:3, SM d18:1/24:2, SM d41:1, SM d14:0/24:1, SM d36:1, Cer m18:1/18:2, Cer d17:1/18:0, GD3 d38:2, GM1 d36:2, GM2 d34:5, GM3 d36:1, GM1 d36:1. Concentration of SM d18:1/18:4 in the DH positively correlated with anxiety level measured in the open field test, but negatively correlated with depression‐like behavior in the tail suspension test. In the PFC of stressed mice, reduced levels of ceramide‐1‐phosphate, SM, SM‐phytosphingosine, GD2, GM3, and GD3 were found. Massive reduction in the concentrations of SM species (SM d44:2, SM d32:1, SM d18:0/22:1, SM d18:1/24:2, SM d18:1/18:4, SM d18:1/23:0, SM d33:1, SM d34:2, SM d34:1, SM d38:0, SM d36:2, SM d41:1, SM d34:0, SM d35:1, SM d18:1/23:3, SM d37:1) was observed in the PFC, while only the amount of Cer m44:3 was significantly enhanced in CUS‐exposed male mice (Zhou et al. 2023; Table 1).

An increase in ceramide load in distinct brain structures may have effects on the emotional state. Gulbins et al. (2013) showed that intracranial administration of Cer16:0 in the DH of mice resulted in depression‐like behavior. Genetically induced ASM overexpression also diminished the rates of neuronal proliferation, maturation, and survival commonly associated with depression‐like behavior (Gulbins et al. 2013). Another study confirmed that ceramide Cer16:0 induced a depressive‐like phenotype when infused into the DH, whereas Cer16:0 induced a predominantly anxiogenic‐like phenotype when infused into the basolateral amygdala of male mice. These effects were not mediated by the changes in the tissue levels of classical neurotransmitters: dopamine (DA), serotonin (5‐HT), and noradrenalin (NA). Ceramides Cer20:0 and Cer8:0 did not have effects on depression‐ or anxiety‐like behavior when injected in the DH and basolateral amygdala (Zoicas, Huber, et al. 2020). However, when injected in the VH, Cer20:0 induced anhedonia‐like behavior in the sucrose preference test, but not depression‐like behavior in the forced swim or sucrose grooming tests (Sambolín‐Escobales, Feliciano‐Quiñones, et al. 2022). Overall, an increase of distinct ceramide species in single brain structures might have brain region‐specific depressogenic and anxiogenic effects.

Enzymes of the SL synthesis were shown to contribute to the pathogenesis of affective disorders. A study of Kornhuber et al. (2005) showed elevated ASM enzyme activity in cultured peripheral blood cells of MDD patients compared to healthy controls (Kornhuber et al. 2005). Analysis of alternatively spliced ASM isoforms in MDD patients showed a reduction in the frequency of ASM alternative splicing events in peripheral blood cells. Moreover, the fraction of the full‐length transcript ASM‐1 was increased in MDD patients, which could result in an increased amount of enzymatically active ASM enzyme derived from ASM‐1 transcripts, leading to elevated catalytic activity levels (Rhein et al. 2017). However, another study showed that serum ASM activity positively correlate with depression severity only in remitted patients, but not in MDD patients at the admission or during treatment (Mühle et al. 2019). Several classical antidepressants, such as amitriptyline, desipramine, and fluoxetine, were shown inhibit ASM activity (Albouz et al. 1986; Kornhuber et al. 2008, 2011). These drugs are suggested to act indirectly via lysosomal trapping of ASM (Kölzer et al. 2004) and are called ‘functional inhibitors of ASM activity’ (FIASMAs) (Kornhuber et al. 2010, 2013). Classical antidepressant drugs, paroxetine and desipramine, reduce total level of ceramides as well as mRNA levels of ASM and AC in the hippocampus, but not in the PFC and striatum of naïve male rats (Jaddoa et al. 2020). In a study on cortical murine neurons, ASM activity and *Smpd1* mRNA expression were reduced by fluoxetine. However, *Smpd1* mRNA expression returned to control levels after 24 h, while ASM activity became even lower. In primary human blood cells, ASM activity and *SMPD1* mRNA expression were reduced both 24 and 48 h after fluoxetine stimulation. In line with human studies, long‐term application of amitriptyline resulted in a decreased *Smpd1* mRNA expression level in the DH of a genetically modified mouse line with the whole‐body ASM overexpression (ASMtg). However, amitriptyline did not affect this parameter in wt mice. Thus, the effects of FIASMAs on *Smpd1* transcription appear to be dependent on the basal ASM level or pathologic conditions (Rhein et al. 2021). Direct inhibition of ASM using a newly developed inhibitor also improved depression‐ and anxiety‐like behavior as shown in male mice in the tail suspension, forced swim, sucrose preference and open field tests. The antidepressant effects of those ASM inhibitors are mediated by multiple depression‐associated mechanisms including effects on apoptosis, levels of BDNF and pro‐inflammatory cytokines, oxidative stress, and serotonin levels (Shi et al. 2024). ASM might be involved in several mechanisms of depression. In particular, depression‐like behaviors and associated ASM overexpression induced by overexpression of the transporter protein sortilin in the PFC and hippocampus of mice was mitigated by injection of the ASM inhibitor, SR33557, into these brain structures. SR33557also restored the loss of dendritic spines induced by specific sortilin overexpression, indicating that the sortilin‐ASM pathway is critical for the development of both pathogenic and behavioral mechanisms underlying depression (Chen et al. 2022). Therefore, inhibition of ASM activity might serve as new target for depression therapy, independent on the mechanism of action of the ASM inhibitors. Another therapeutic approach for depression, namely psychosomatic‐psychotherapeutic treatment, was shown to reduce symptom severity of depression, anxiety, and somatization as well as cortisol levels. It also normalized the molar ratio of ceramide species Cer16:0 and Cer18:0, whereas Sph and S1P levels increased. However, enzymatic activities of secreted ASM, NSM, and NC increased significantly upon behavioral treatment. The authors of this study suggest that the enzymatic rheostat mediating ceramide metabolism might be disturbed in stress‐associated diseases and is getting rebalanced by treatment into the direction of Sph and S1P (Werner et al. 2025).

Mouse lines that overexpress ASM and are heterozygous for AC exhibit depression‐like behavior, which is constitutively associated with diminished hippocampal neurogenesis (Gulbins et al. 2013; Müller et al. 2017). The antidepressants amitriptyline and fluoxetine were found to decrease ASM activity in the DH of mice, to rescue neuronal proliferation, maturation, and survival of hippocampal neurons, and to improve stress‐related depression‐like behavior in mice. In contrast, ASM knockout mice showed reduced depression‐like behavior. Antidepressant drugs had no effect on emotional phenotype or hippocampal ceramide abundance in these mice (Gulbins et al. 2013). Selective ASM overexpression in the forebrain of male mice was also associated with a depressive‐like phenotype accompanied by an increase in Cer24:0 amount in the DH and a decrease in Cer18:0 concentration in the VH. In females, however, forebrain ASM overexpression resulted in a social anxiogenic‐like phenotype (Zoicas, Schumacher, et al. 2020).

Female rats selectively bred for high anxiety‐like behavior (HAB) showed a brain‐region specific increased activity of ASM, NSM, AC, and NC in multiple brain regions associated with anxiety‐ and depressive‐like behavior compared to female rats selectively bred for low anxiety‐like behavior (LAB). HAB females have higher ASM activity in the lateral septum, hypothalamus, VH, and ventral mesencephalon compared with LAB females, whereas no differences in the activity of this enzyme were found in the frontal cortex (FC), amygdala, DH, dorsal and ventral striatum (DS and VS), dorsal mesencephalon, thalamus, and cerebellum. The activity of NSM was enhanced in HAB female rats only in the ventral mesencephalon. HAB females also showed an increased AC activity in the dorsal and VS, hypothalamus, thalamus, and ventral mesencephalon. Increased NC activity was observed in the hypothalamus and dorsal mesencephalon of HAB females, while the NC activity in the amygdala was reduced. Anxiety‐like behavior in HAB females negatively correlated with ASM activity in the amygdala, VH, and dorsal mesencephalon and with NSM activity in the dorsal mesencephalon, but with none of the parameters in LAB females. In turn, depressive‐like behavior negatively correlated with AC activity in the FC in LAB females, but not in HAB rats (Zoicas, Mühle, et al. 2020).

Recent studies indicate that depressogenic effects might be mediated by the peripheral pool of ceramides. In two studies by Schumacher, Carpinteiro, et al. (2022); Schumacher, Edwards, et al. (2022), treatment of female mice with the stress hormone glucocorticosterone or exposure to CUS, which were associated with the development of depression‐like behavior, resulted in an increase in the plasma total ceramide concentration as well as specific levels of Cer22:0, Cer24:0, and Cer24:1. Interestingly, loading the blood plasma of naive mice with the ceramide mixture (a ratio of 5.2%: 2.4%: 2.5%: 13.6%: 55.5%: 20.8% of Cer16: Cer18: Cer20: Cer22: Cer24: Cer24:1) or intravenous injection of ceramide‐loaded exosomes was sufficient to induce depression‐like behavior and reduce neuronal proliferation. Peripheral administration of ceramides resulted in subsequent ceramide accumulation in hippocampal endothelial cells, but not whole hippocampal tissue. This accumulation was accompanied by a marked reduction of phospholipase D (PLD) and phosphatidic acid concentrations. Moreover, treatment with anti‐ceramide antibodies, ceramidase, or phosphatidic acid normalized depression‐like behavior and reduced neurogenesis in CUS exposed female mice. These data indicate that a depressive state could also be induced by an increase in peripheral ceramides via the PLD‐phosphatidic acid system of the hippocampal endothelial cells. Therefore, blockage of accumulation of peripheral ceramides might have protective effects against the development of depression‐like behavior (Schumacher, Carpinteiro, et al. 2022; Schumacher, Edwards, et al. 2022).

Another type of sphingolipids, gangliosides, also mediate the emotional state of an individual. For instance, in a model of Tay‐Sachs disease, a neurodegenerative lysosomal storage disorder related to GM2‐gangliosidosis, male mice showed an anxiety‐like phenotype and high levels of GM2 ganglioside in cortical regions. This is associated with elevated neuroinflammation and a significant loss in neuronal density and oligodendrocytes (Demir et al. 2020). In line, long‐term intraventricular administration of GM1 resulted in a reduction in depression‐ and anxiety‐like behavior across mouse models of Huntington disease with different genetic backgrounds (Table 7). Exogenous GM1 retarded neurodegeneration and white matter atrophy and modulated neurotransmitter metabolism in the brains of these mice (Alpaugh et al. 2017). Conversely, a decrease in C18:0 ganglioside levels by CerS1 ablation in mice blocks the susceptibility to develop anxiety‐like behavior (Ginkel et al. 2012). Knockout mice with a deficiency in GD3 and the downstream b‐series gangliosides, including GD3, GD2, GD1b, GT1b, and GQ1b, are also characterized by increased immobility time and decreased latency to immobility in tail suspension and forced swim tests. This depression‐like phenotype was associated with impaired neurogenesis in the granular cell layer of the olfactory bulb and the dentate gyrus of adult GD3 knockout mice. Importantly, the self‐renewal capacity of neural stem cells and radial glia‐like stem cell outgrowth in postnatal GD3 knockout could be rescued by the restoration of GD3 expression. It should be emphasized that these effects develop only in adulthood, as embryonic neurogenesis was to a big extent preserved. Therefore, GD3 and the downstream b‐series gangliosides determine maintenance, rather than the generation of the neural stem cells (Wang et al. 2014).

Altogether, several members of the SL family were shown to control emotional phenotype in humans and rodents and may essentially contribute to stress resilience. Moreover, some principally new SL‐associated mechanisms of affective disorders have been recently proposed, indicating high potential of further investigation of SLs as targets and markers of emotional well‐being.

### 3.2 Sphingolipids in Cognitive Performance

The efficient and successful adaptation of living organisms to their environmental conditions is largely contingent upon the integration and processing of information from both external stimuli and internal physiological signals. Cognition is a complex multidimensional capacity, which includes the ability to learn, retain, and recall information as well as the ability to perform judgments, evaluations, and predictions based on previous experience (Luine 2014). Eudemonic aspects of well‐being, which refer to a sense of purpose and meaning in life (Waterman et al. 2010), are associated with better performance on cognitive tests (Welch and West 1995; West et al. 2008), while cognition very slightly affects hedonic aspects of positive affect or life satisfaction (Wilson et al. 2013). This is especially important for older individuals, where a better cognitive performance in terms of processing speed and spatial ability is associated with higher scores on life satisfaction (Enkvist and Elmståhl 2013). Volunteers with better cognitive performance tend to experience smaller impairments of positive mood and smaller increases in negative mood after exposure to stressors, suggesting higher emotional resilience to daily stress (Stawski et al. 2010). Altogether, cognitive performance and cognitive flexibility might play an important role in the maintenance of emotional well‐being. In turn, cognition has been recently shown to be mediated by SLs. These lipid classes contribute to the de novo mechanisms of memory, but also determine the pathogenesis of cognitive decline.

The SL system, and particularly enzymes of ceramide synthesis, were shown to contribute to the mechanisms of de novo learning and memory. Kalinichenko, Abdel‐Hafiz, et al. (2021) showed that in healthy male human volunteers, an enhanced NSM activity predicted superior appetitively motivated long‐term logical memory in the Rivermead Behavioral Memory Test. It can be concluded that NSM might serve as a marker of certain types of cognitive performance. The specific role of NSM in the mechanisms of appetitively and aversively motivated memory was also demonstrated in different mammalian species. In naïve male Wistar rats, better performance in tests assessing short‐term and long‐term memory, such as spontaneous alternation and novel object recognition test, was associated with higher NSM activity as well as enhanced levels of SM18:0 and Cer18:0 in the VS and DH (Table 2). Other SM or ceramide species were not altered under these conditions. A similar pattern was found in male non‐human primates (Callinix penicillate). A specific increase in serum NSM, but not ASM activity, was predictive for better memory performance in the object discrimination test. The authors suggested NSM activity in the serum as a potential marker of changes in the brain SL metabolism associated with cognitive performance in various mammalian species and humans (Kalinichenko, Abdel‐Hafiz, et al. 2021). A study performed on genetically modified mice with NSM hypoexpression (Fro mice) confirmed this proposal, in a sex‐specific way. A reduction in NSM activity in naïve female, but not male, Fro mice was associated with worse memory performance in the spontaneous alternation test and novel object recognition test. A different pattern of associations was found in tests for aversive memory performance. Naïve male Wistar rats with bad learning abilities in the Morris water maze test were characterized by higher NSM activity in the DS. Interestingly, high NSM activity did not predict changes in SM and ceramide levels in the DS in bad learners in the short‐term version of the test. On the contrary, bad learners in the short‐term version of the test had high NSM activity as well as high SM18:0 level in the DS. Similar, reduced NSM activity in male heterozygous Fro mice was associated with enhanced spatial memory in the Morris water maze test. A moderate impairment in the aversive memory performance in this test was observed in female Fro mice (Kalinichenko, Abdel‐Hafiz, et al. 2021). However, pharmacological inhibition of NSM in C57BL6 male mice by GW4869 diminished learning in the Morris water maze test and radial arm maze. However, NSM inhibition did not abolish spatial learning, as memory persisted in the subsequent repeated reversals tasks. Therefore, NSM‐dependent plasticity might be crucial for procedural learning, required for the acquisition in the Morris water maze, but may not contribute to the mechanisms of episodic‐like memory, required for repeated reversals tasks (Tabatadze et al. 2010). In line, associative aversive learning in the contextual fear‐conditioning learning test was also improved in male Fro mice with full knockout of NSM. In contrast, a knockout of NSM in 5XFAD mice was associated with a significant reduction in total brain ceramides Cer16:0, Cer18:0, Cer24:1, and Cer18:1 and improved cognition in a fear‐conditioned learning task, compared to 5XFAD mice (Dinkins et al. 2016).

**TABLE 2:** Sphingolipids in cognitive performance: Associations between memory performance and tissue levels of ceramides and sphingomyelins.

Subject	Changes in memory performance	Changes in SL levels	Tissue	References
Preclinical studies				
Male rats	↑ performance in spontaneous alternation and novel object recognition test	↑ Cer18:0 ↑ SM18:0	Ventral striatum and dorsal hippocampus	Kalinichenko, Abdel‐Hafiz, et al. 2021
5XFAD male mice with NSM knockout	↑ performance in fear‐conditioned learning task	↓ Cer16:0, Cer18:0, Cer24:1, and Cer18:1	Total brain	Dinkins et al. 2016
CerS6 knockout male mice	↓ open field behavioral habituation	↓ Cer16:0	Forebrain, cerebellum, small intestine, thymus, and kidney	Ebel et al. 2013
Aged male rats	↓ performance in the hole‐board test	↑ Cer17:2 and Cer19:2	Hippocampus	Wackerlig et al. 2020
Obese male rats	↓ novel object recognition test	↑ Cer d18:1/16:0, Cer d18:1/18:0, Cer d18:1/22:0 ↑ HexCer d18:1/24:0 ↑ LacCer d18:1/24:0 ↑ SM d18:1/14:0, SM d18:1/22:0, and SM d18:1/24:0	Hippocampus and frontal cortex	Santillán et al. 2025
Clinical studies				
Elderly females	↓ memory performance or delayed recall in the Hopkins Verbal Learning Test ↓ psychomotor speed	↓ Cer16:0, Cer20:0, and stearoyl ceramide	Serum	Mielke, Haughey, et al. 2010
Elderly females and males	↓ risk of Alzheimer's disease‐associated dementia	↑ ratio of Cer22:0/Cer16:0 and Cer24:0/Cer16:0	Serum	McGrath et al. 2020

Abbreviations: Cer, ceramide; HexCer, hexosylceramide; LacCer, lactosylceramide; SM, sphingomyelin.

ASM is another enzyme of the sphingomyelinase pathway of ceramide synthesis, which contributes to the mechanisms of de novo learning and memory. A specific increase in ASM activity in the VS, but not other brain structures of naïve Wistar rats, was associated with superior short‐term memory performance in the spontaneous alternation test. ASM activity was not predictive for memory performance in the novel object recognition test and Morris water maze test. In male non‐human primates (Callinix penicillate), serum ASM activity also did not predict object learning and memory performance in the novel object recognition test. However, in healthy male human volunteers, higher serum ASM activity predicted better logical memory performance as tested in the RBMT (Kalinichenko, Abdel‐Hafiz, et al. 2021). Another type of memory, behavioral extinction, was also shown to be ASM‐dependent. A specific decrease in the activity of ASM, but not other enzymes of this pathway of ceramide synthesis, in the DH of Wistar rats, was associated with the learning‐related measures of behavioral extinction. Stronger reduction in ASM activity in this brain structure predicted a more rapid extinction. The decrease in ASM activity was associated with a general decline in ceramide, but not SM levels in the brain, which was mostly driven by the long chain ceramide Cer16:0 in the DH (Huston et al. 2016). Zoicas et al. (2016) did not observe changes in novel object recognition and object discrimination indicating intact short‐term non‐social memory abilities in both male and female mice overexpressing ASM. However, amitriptyline treatment impaired both novel object recognition and object discrimination in female wt, but not transgenic mice, indicating that ASM overexpression protected female mice against the detrimental effects of amitriptyline on non‐social memory (Zoicas et al. 2016). In conditioned place preference (CPP) test with alcohol treatment, memory performance of mice with genetically‐induced ASM overexpression did not differ from wt littermates. However, ASM deficiency reduced the speed of learning of rewarding properties of alcohol in a mixed male and female cohort (Müller et al. 2017). Altogether, both ASM and NSM contribute to the mechanisms of de novo learning and memory and might serve as possible markers of memory performance in a sex‐specific way.

Ceramidases are another group of enzymes of the sphingomyelinase pathway of ceramide degradation. A study by Kalinichenko, Abdel‐Hafiz, et al. (2021) showed a predictive potential of high NC activity in the blood serum of naïve male rats and in non‐human primates for learning and memory performance in a long‐term novel object recognition task. In line, better working memory performance in a spontaneous alternation test was associated with enhanced NC activity in the ventral mesencephalon, but not in other brain structures of rats. Interestingly, AC activity was not found to be predictive for any type of aversive or appetitive, object or spatial, short‐ or long‐term memory in this study (Kalinichenko, Wang, et al. 2021).

SMS are enzymes responsible for sphingomyelin synthesis from ceramide. A knockout of SMS2 in mice was associated with impaired memory performance in the Morris water maze test. However, the cognitive performance of SMS2 knockout mice in the context‐dependent fear learning and novel object recognition tests was preserved (Wang et al. 2017).

The catabolic pathway of ceramide synthesis mediated by ceramide synthases also contributes to the de novo memory formation. Particularly, a study by Ebel et al. (2013) showed a crucial role of CerS6 in spatial information gathering. A knockout of the CerS6 gene results in a reduced level of Cer16:0, but not other SLs in the forebrain, cerebellum, and several peripheral tissues and a deficiency of behavioral habituation to a novel environment. However, the short‐term object memory in the object recognition test was intact in these mice (Ebel et al. 2013).

Ceramide transfer proteins (CERTs) are ceramide carriers. Neuronal overexpression of CERTs in a mouse model of familial Alzheimer disease (5xFAD) was not associated with changes in working and intermediate spatial memory performance measured in the Y‐maze spontaneous alternation test, despite changes in the ceramide composition of the brain (Crivelli et al. 2021).

Converging evidence now implicates an important role of SLs in the development of cognitive decline under certain physiological conditions, such as aging. A cognitive decline associated with the normal aging process and occurring in the absence of disease might diminish emotional well‐being (Enkvist and Elmståhl 2013). Older individuals display a slower processing speed, diffuse attention, and difficulties with learning new information (Dinius et al. 2023). Decline in memory performance or delayed recall in the Hopkins Verbal Learning Test and reduced psychomotor speed was associated with a low level of total ceramides and SMs, and particularly serum concentrations of the long‐chain ceramides Cer16:0, Cer20:0, and stearoyl ceramide in elderly females. In turn, higher levels of these sphingolipids predicted this impairment for up to 9 years (Mielke, Haughey, et al. 2010). Preclinical studies also show pronounced changes in the SL balance during aging. Twenty‐four‐month‐old rats were characterized by a higher concentration of total ceramide and reduced level of total SM in the serum, liver, heart, soleus muscle, and gastrocnemius muscle compared to 3‐month‐old rats (Bárcena et al. 2021). Ceramide species‐specific changes were also associated with aging. An increase in the very long‐chain ceramide Cer24:1 in the extracellular vesicles of older monkeys, and ceramide Cer24:0 and GalCer24:0 in the cerebral cortex of aged C57BL/6 mice were shown. In line, aging was associated with a decrease in SM24:0 level (Khayrullin et al. 2019). The concentration of long‐chain ceramides Cer17:2 and Cer19:2 was also enhanced in the hippocampus of 20‐month‐old rats compared to young animals. This increase was associated with impairment in spatial memory as tested in the hole‐board test (Wackerlig et al. 2020). Obesity‐associated impairment of object memory as measured in the novel object recognition test in rats was accompanied by increased concentration of ceramides Cer d18:1/16:0, Cer d18:1/18:0, Cer d18:1/22:0, HexCer d18:1/24:0, and LacCer d18:1/24:0 and sphingomyelins SM d18:1/14:0, SM d18:1/22:0, and SM d18:1/24:0 in the hippocampus and FC. Interestingly, changes in the brain SL system were observed even in the offspring of obese parents. However, enhanced expression of SPT1 and CerS 2 in the hippocampus and FC of F1 rats born to obese dams was not associated with changes in learning abilities (Santillán et al. 2025; Table 2). The specific role of the sphingomyelinase, but not other pathways of ceramide synthesis, in the age‐dependent memory decline should be emphasized. Park et al. (2018) observed protective effects of the ASM on the age‐dependent decline of spatial memory. As distinct from wt mice, old *Smpd1* heterozygous mice with reduced ASM activity were shown to be protected from the cognitive decline observed in the Morris water maze test. However, diminished ASM activity did not affect in long‐term potentiation pattern in old mice (Park et al. 2018).

Cognitive decline often accompanying neurodegenerative disorders, such as Alzheimer's disease, are also associated with changes in the ceramide system. High serum or plasma levels of ceramides, such as Cer16:0, Cer20:0, Cer24:0, stearoyl ceramide, or LacCer might predict the risk of memory impairments including verbal memory impairments, cross‐sectional memory impairment on delayed recall in the Hopkins Verbal Learning Test, immediate recall and psychomotor speed in the Trail Making Test (Mielke et al. 2015) or all‐cause dementia (Mielke et al. 2012). This association was shown to be most significant in female patients. A study by McGrath et al. (2020) revealed that an increase in the ratio of ceramides Cer22:0/Cer16:0 and Cer24:0/Cer16:0 is associated with a significant reduction in the risk of Alzheimer's disease‐associated dementia in elderly patients of both genders (McGrath et al. 2020). Moreover, an increase in the plasma levels of Cer16:0, Cer18:0, and Cer20:0 was associated with hippocampal atrophy in patients with Alzheimer's disease, which were younger than 75 years (Kim et al. 2017). Therefore, the ceramide system might serve as a predictor of certain types of memory impairments under physiological and pathological conditions. It should be emphasized that these associations are highly dependent on the ceramide species, gender, and memory type. Targeting the SL system could be proposed as potential therapeutic approach for treatment of cognitive decline. For example, feeding of preterm low‐birth‐weight babies with SM‐fortified milk improved the developmental prognosis, enhanced Behavior Rating Scale of the Bayley Scales of Infant Development (BSID‐II) indicating infants' mental and psychomotor development, the Fagan test scores indicating visual recognition memory, the latency of the visual evoked potentials, and sustained attention test scores at 18 months compared to controls (Tanaka et al. 2013).

Very few studies focused on the involvement of gangliosides in de novo memory performance. GD3 synthase knockout mice, characterized by the lack of GD3 as well as other b‐series gangliosides, such as GD2, GD1b, GT1b, and GQ1b, have impaired spatial and non‐spatial hippocampus‐dependent memory function, as measured in the Barnes maze and in the novel object recognition tests (Tang et al. 2021; Table 7). Male, but not female, GM3 synthase knockout mice also show impaired working memory in the spontaneous alternation Y‐maze test (Chowdhury et al. 2023; Niimi et al. 2013). In line, adult GM2/GD2 synthase knockout mice, with a lack of GA1, GA2, GM1b, GD1b, and GD1c gangliosides, showed impaired aversive spatial memory in the Morris water maze test and aversive non‐spatial memory in the step‐down inhibitory avoidance task (Sha et al. 2014). Double knockout of GM2/GD2 synthase and GD3 synthase genes in mice is also associated with a decline in spatial learning and memory in the 8‐arm radial maze, both in young and old mice (Tajima et al. 2010). Similar, non‐specific inhibition of ganglioside synthesis with D‐threo‐1‐phenyl‐2‐decanoylamino‐3‐morpholino‐1‐propanol (D‐PDMP) in mice resulted in a failure of learning in the 4‐pellet taking test for spatial memory, while stimulation of the synthesis by L‐PDMP did not have any effects (Fujiwara et al. 2012). These data indicate that non‐specific reduction in ganglioside composition results in a cognitive decline. However, the treatment of mammals with exogenous gangliosides is widely shown to improve various types of memory, such as spatial, aversive, and working memory (Liu et al. 2014; Silva et al. 2000; Jung et al. 2008). Development and aging are widely associated with alterations in ganglioside composition in biological tissues. The embryonic brain is enriched in simple ganglioside forms, such as GM3 and GD3, while more complex forms, GM1a, GD1a, GD1b and GT1b, for which GM3 and GD3 are precursors, are abundant only in the adult brain (Yu et al. 2012; Ngamukote et al. 2007). Aging is further associated with an increase in the levels of GQ1b, GT1b, and GD1b and a concomitant decrease in GM1a and GD1a (Palmano et al. 2015). It should be emphasized that the age‐related changes in the brain ganglioside composition are brain structure‐dependent. Particularly, an age‐related increase in the Cer20‐GD1 was found selectively in the dentate gyrus molecular layer and the stratum lacunosum moleculare of both CA1 and CA3, but not in other hippocampal regions of aged male C57BL/6Cr mice. In turn, the concentration of Cer18‐GD1 in this brain area decreased with aging. These changes might be responsive to the reduced membrane fluidity in older individuals, as Cer20‐GD1 causes a fluidity drop as compared to Cer18 species (Sugiura et al. 2008). Interestingly, ganglioside replacement therapy is effective against cognitive impairments during aging and under pathological conditions, for example, in patients with Alzheimer's disease, as shown in preclinical and clinical studies (Fong et al. 1997; Silva et al. 1996; Jeon et al. 2016; Svennerholm et al. 2002; Yang et al. 2013; Shin et al. 2019; Fujiwara et al. 2012).

Altogether, this growing body of evidence suggests that SLs are involved in the mechanisms of cognitive performance in healthy and diseased individuals. Although further analysis on the SL species‐specificity of these lipid processes is of specific interest, current evidence suggests that modulation of cognitive performance is likely one of the key pathways through which SLs contribute to overall well‐being.

### 3.3 Sphingolipids in Social Behavior

Social behavior, including social interaction, communication and affiliative bonding, is fundamental to psychological and physiological well‐being in both humans and rodents. It underlies emotional regulation, stress resilience and cognitive function (Schäfer et al. 2024; Xia et al. 2025; McManus et al. 2020; Beery and Kaufer 2015). Deprivation or dysregulation of social contact, whether through isolation or overstimulation, can profoundly disrupt mental health, often leading to mood disturbances and behavioral instability (Benke et al. 2020; Lincoln 2000). These effects are reflected in both clinical populations and animal models: impaired social functioning is a defining feature of numerous psychiatric and neurodevelopmental disorders, including autism spectrum disorder (ASD), social anxiety disorder, MDD and avoidant personality disorder. In rodent models, social stress consistently elicits behavioral phenotypes such as social withdrawal, increased anxiety‐ and depression‐like behaviors and cognitive decline (Toth and Neumann 2013; Beery and Kaufer 2015), underscoring the evolutionary conservation of social behavior circuits and their vulnerability to disruption.

Converging evidence now implicates a central role of SLs in shaping social interaction and emotional resilience. In humans, particularly in the context of ASD, aberrant SL profiles have been reported. Post‐mortem analysis of individuals with autism revealed elevated ceramide levels in the PFC (Yu et al. 2020), a region integral to social cognition and decision‐making. Blood‐based metabolomic studies have identified differential associations between ASD and specific SM species, such as SM d17:1/16:0 and SM d18:1/20:1, indicating potentially causal lipidomic imbalances (Li et al. 2024). Cerebrospinal fluid analyses from children with autistic regression show widespread disturbances in SL metabolism, including elevated levels of various ceramides, for example, Cer d18:1/16:0, Cer d18:1/16:1, Cer d18:1/18:0, Cer d18:1/20:0, Cer d18:1/20:1, Cer d18:1/22:0, Cer d18:1/24:0, Cer d18:1/24:1, hexosylceramides, for example, HexCer d18:1/16:0, HexCer d18:1/18:0, HexCer d18:1/24:1, HexCer d18:2/22:0, HexCer d18:2/24:1, sphingosines, Sph 18:0, Sph 18:1, and S1P, alongside reduced levels of key SMs like SM d18:1/16:0 and SM d18:1/18:0 (Yan et al. 2025; Table 3). These findings suggest not only a disruption in membrane lipid homeostasis but also potential imbalances in signaling molecules that modulate neuroplasticity and inflammation (Arsenault et al. 2021; Gomez‐Larrauri et al. 2025; Olsen and Færgeman 2017).

**TABLE 3:** Sphingolipids in social behavior: Changes in tissue levels of ceramides and sphingomyelins in preclinical and clinical studies.

Preclinical studies				
Model	Species	Changes	Tissue	References
Chronic social defeat stress	Male mice	↑ total Cer, SM ↓ total HexCer, S1P and Sph	Hippocampus	DeVeaux et al. 2024
		↓ total Cer, SM ↑ total HexCer, S1P and Sph	Cortex	
Social isolation	Male mice	↑ SM24:0	Dorsal hippocampus	Zoicas et al. 2023
		↓ Cer24:1, SM22:0, SM24:1, and Sph	Ventral mesencephalon	
	Male dogs	↑ total LacCer, GluCer, GalCer, and SM ↑ LacCer d18:1/22:0 ↑ GluCer d18:1/18:0, GluCer d18:1/24:0, GluCer d18:1/24:1, GluCer d18:1/26:1 ↑ GalCer d18:0/25:1, GalCer d18:0/25:0, GalCer d18:0/26:1	Cerebrospinal fluid	Hong et al. 2023
Social fear conditioning	Male mice	↓ Cer16:0, Cer18:0, Cer22:0 and total ceramide	Frontal cortex	Zoicas et al. 2023, 2025
		↓ SM16:0, SM18:0, Cer18:0, Cer20:0, Cer22:0, Cer24:0 and total Cer ↑ SM24:1	Thalamus	
		↓ Sph ↑ SM24:1	Dorsal hippocampus	
		↑ SM18:0	Dorsal mesencephalon	
		↑ SM20:0	Ventral mesencephalon	

Clinical studies				
Disorder	Gender	Changes	Tissue	References
Autism	Males and females	↑ total Cer	Prefrontal cortex	Yu et al. 2020
Autistic regression	Males and females	↑ Cer d18:1/16:0, Cer d18:1/16:1, Cer d18:1/18:0, Cer d18:1/20:0, Cer d18:1/20:1, Cer d18:1/22:0, Cer d18:1/24:0, Cer d18:1/24:1 ↑ HexCer d18:1/16:0, HexCer d18:1/18:0, HexCer d18:1/24:1, HexCer d18:2/22:0, HexCer d18:2/24:1 ↑ Sph 18:0, Sph 18:1, S1P ↓ SM d18:1/16:0 and SM d18:1/18:0	Cerebrospinal fluid	Yan et al. 2025

Abbreviations: Cer, ceramide; GalCer, galactosylceramide; GluCer, glucosylceramide; HexCer, hexosylceramide; LacCer, lactosylceramide; S1P, sphingosin‐1‐phosphat; SM, sphingomyelin; Sph, sphingosine.

An intriguing and somewhat paradoxical observation concerns GM1 ganglioside. On one hand, elevated levels of GM1 and other major gangliosides (e.g., GD1a, GD1b, GT1b) have been reported in the cerebrospinal fluid (CSF) of children with ASD (Lekman et al. 1995), and increased GM1 content has been detected in erythrocyte membranes of autistic children (Schengrund et al. 2012). On the other hand, elevated titers of anti‐GM1 autoantibodies in serum, plasma and CSF have been documented in autistic patients and correlate with symptom severity (Mostafa and AL‐ayadhi 2011; Hamed et al. 2022; Ashaat et al. 2023). These autoantibodies may represent an aberrant immune response that compromises GM1's neuroprotective function, despite increased levels. This dysfunction contrasts with rodent studies, where exogenous GM1 administration robustly improves social behavior. In chronic social defeat stress models, a well‐established model of social withdrawal and depression‐like behavior (Slattery and Cryan 2017; Toth and Neumann 2013), daily intraperitoneal GM1 injections reverse social avoidance and anhedonia and restore BDNF signaling in the hippocampus and medial PFC (Jiang et al. 2016; Table 7). Similarly, in valproic acid‐exposed rats, a well‐established ASD model (Nicolini and Fahnestock 2018), GM1 treatment improves social interaction, cognition and repetitive behaviors (Yin et al. 2023). This dichotomy suggests that endogenous GM1 may be functionally compromised in ASD, possibly due to autoantibody interference or mislocalization, whereas pharmacological GM1 supplementation may bypass these pathological barriers and restore key neurotrophic functions.

Rodent models further support a causal relationship between SL dysregulation and social dysfunction. Transgenic mice overexpressing ASM exhibit reduced social exploration, increased anxiety‐ and depression‐like behaviors and elevated hippocampal ceramide levels (Gulbins et al. 2013; Zoicas et al. 2016; Müller et al. 2017). Targeted overexpression of ASM in the forebrain produces similar behavioral effects, with region‐specific changes in ceramide composition, for example, elevated Cer24:0 in the DH and reduced Cer18:0 in the VH, suggesting that a subregional SL balance is critical for maintaining healthy social behavior (Zoicas, Schumacher, et al. 2020).

These region‐ and state‐dependent alterations are further illustrated in the social fear conditioning (SFC) paradigm, a validated animal model of social anxiety disorder (Kornhuber and Zoicas 2019; Kornhuber and Zoicas 2020). In the early phase of SFC‐induced psychopathology, when social fear is the only observed symptom (Toth et al. 2012; Zoicas et al. 2023), increased AC and NC activity was found in brain regions such as VH and ventral mesencephalon, alongside elevated SM24:1 levels in the DH (Zoicas et al. 2023). As social fear progresses into a depressive‐like phenotype, broader SL disruptions emerge, with reductions in ceramide and Sph across multiple brain regions (FC, thalamus, hippocampus), coupled with selective elevations in SM levels in mesencephalic structures (Zoicas et al. 2025; Zoicas et al. 2023). More specifically, reduced levels of Cer16:0, Cer18:0, Cer22:0 and total ceramide are observed in the FC; reduced SM16:0, SM18:0, Cer18:0, Cer20:0, Cer22:0, Cer24:0 and total ceramide are found in the thalamus and reduced Sph is detected in the DH. In contrast, SM24:1 levels were elevated in the thalamus, while SM18:0 and SM20:0 showed marked increases in the dorsal and ventral mesencephalon, respectively (Table 3). These transitions suggest that distinct SL signatures may underlie different stages or facets of social anxiety disorder‐related psychopathology.

Complementary patterns have been reported in other models of social dysfunction. Chronic social defeat stress leads to increased ceramide and SM levels, and decreased HexCer, S1P, and Sph in the hippocampus, while the cortex shows a reciprocal shift (DeVeaux et al. 2024), indicating region‐specific lipidomic remodeling in response to chronic social stress. Prolonged social isolation in mice reduces activities of ASM, NSM, AC, and NC in key socio‐emotional brain centers, including the hypothalamus, amygdala, and ventral mesencephalon, and disrupts regional SL balance (Zoicas et al. 2023). Specifically, socially isolated mice exhibit increased SM24:0 levels in the DH, while decreased levels of SM22:0, SM24:1, Cer24:1, and Sph were observed in the ventral mesencephalon (Zoicas et al. 2023). In canine studies, total isolation from both humans and conspecifics for 4 weeks resulted in elevated cerebrospinal fluid levels of polar SLs, including LacCer, glucosylceramide, GalCer, and SM (Hong et al. 2023), supporting a conserved role of SLs in social behavior across mammalian species (Table 3).

In ASD‐specific rodent models such as the 16p11.2 deletion mouse (Portmann et al. 2014; Wang et al. 2018), hallmark behavioral phenotypes such as hyperactivity and impaired social interaction were accompanied by reductions in SM and HexCer in the striatum (Ju et al. 2021), linking SL deficits to behavioral alterations common in ASD.

Together, this growing body of evidence suggests that SLs, particularly ceramides, SMs, HexCers, and gangliosides such as GM1, serve as dynamic regulators of social behavior. Dysregulation in their metabolism may not merely reflect a consequence of psychopathology but could play a causative role in the development of social dysfunction and thus may impact well‐being.

### 3.4 Sphingolipids in Reward Seeking Behavior

One of the crucial components of well‐being, hedonic well‐being, is associated with pleasures and determines a general interest in life (Oltean et al. 2025; Ryff et al. 2004). Overall, a positive outlook on life is associated with attending to rewards and the regular achievement of wished goals (Raila et al. 2015). Reward processing, consisting of learning, interpreting, and responding to a positive stimulus, motivates an individual for certain types of activities as it is associated with positive, hedonic, pleasurable processes (Elliot et al. 2013; Esch and Stefano 2004; Michaelsen and Esch 2021). If approach motivation results in the achievement of the stimulus or goal, a reward is experienced as a pleasurable feeling. Increased reward sensitivity is frequently associated with happiness and positive affect (Taubitz et al. 2015; Dornbach‐Bender et al. 2020), while impaired reward processing is related to diminished subjective well‐being (Gilleen et al. 2015; Whitton et al. 2023). Rewarding stimuli are important not only for well‐being, but also for an organism's survival. Primary rewards are typically crucial for survival and reproduction. However, secondary rewards are acquired through learning and are perceived as beneficial to the organism, such as monetary gain or social interactions. Both types of stimuli induce similar activation of the reward system and share neurobiological mechanisms (Sescousse et al. 2013). In this chapter, we will discuss the role of SLs in reward associated with drug addiction and will focus especially on the controlled use of psychoactive substances not associated with addiction. On one hand, very few literature data on the contribution of SLs to nondrug‐related reward are available so far, while multiple data demonstrate the importance of these lipid molecules for drug‐related behavior. On the other hand, controlled consumption of psychoactive drugs is one of the key mechanisms of modulation of other types of behavior, such as the reduction of stress manifestation or symptoms of affective disorders, enhancement of social and/or sexual behavior, and others (Müller et al. 2023; Müller and Schumann 2011). Therefore, goal‐directed behavior might have not only reward‐associated hedonic pleasure but also enhance other types of behavior leading to an increase in well‐being.

Food is one of the main natural rewards. Multiple studies show the dependence of the SL composition of various tissues on food content, emphasizing the importance of nutrition for lipid‐mediated metabolic and psychiatric processes (Wang et al. 2025; Wang et al. 2023; Sambolín‐Escobales, Tirado‐Castro, et al. 2022; Ordóñez‐Gutiérrez et al. 2021; Babenko and Semenova 2010). Lately, a batch of studies focused on the role of the SL system in the rewarding properties of food and/or reward‐associated behavior. A study of Huston et al. (2016) showed that certain components of food reward‐dependent behavior are mediated by the SL system. Rapid extinction, indicating efficient active re‐learning in the food‐based operant task test, was associated with a decrease in the activity of ASM in the DH of rats. The degree of the decline in ASM activity correlated with the intensity of extinction, thus indicating behavioral flexibility. Authors observed that only the learning‐related measures of extinction were associated with a general decline in total ceramide, but not SM levels in the brain. The most pronounced decrease was observed for Cer16 in the DH and Cer24 in the VH (Table 4). Interestingly, the decline in ceramide concentrations was not accompanied by changes in other enzymes of ceramide metabolism, such as NSM, AC, and NC. Therefore, ASM was proposed as a crucial mechanism of extinction of learned appetitive behaviors (Huston et al. 2016). On the contrary, the rewarding effects of sugar pellets did not differ between ASM overexpressing mice and wt littermates (Müller et al. 2017). In line, no differences in the rewarding properties of a special food preparation with a high fat/carbohydrate ratio and high addictive potential (Hess et al. 2019) were observed in either female or male mice with the forebrain overexpression of ASM (Kalinichenko et al. 2025). NSM was also shown not to be involved in the establishment of the conditioned reinforcing effects of this preferred food (Hess et al. 2019) in males. Therefore, the SL system mediates only certain mechanisms of the natural rewarding system, probably depending on the satiety state.

**TABLE 4:** Sphingolipids in reward seeking behavior: Changes in tissue levels of ceramides and sphingomyelins as a response to different types of reward.

Type of reward	Model	Species	Changes	Tissue	References
Food	Extinction in the food‐based operant task test	Male rats	↓ total Cer ↓ Cer16:0	Dorsal hippocampus	Huston et al. 2016
			↓ total Cer ↓ Cer24:0	Ventral hippocampus	
Alcohol	Alcohol feeding	Male mice	↓ SM16:0, SM18:1, and SM18:0	Serum	Zhao et al. 2011
			↑ SM16:0, SM18:1, and SM18:0	Heart	
			↓ Cer18:0 and Cer16:0	Liver	
			↑ Cer18:0 and Cer16:0	Kidneys	
		Male mice	↑ Cer22:0 and Cer24:0	Liver	Carr et al. 2014
		Male mice	↑ Cer16:0, Cer18:0, and Cer24:0	Liver	Sozio et al. 2010
	Adolescent or prenatal alcohol administration	Male mice	↑ total Cer	Brain	Saito et al. 2007, 2010
	Binge alcohol drinking	Male mice	↓ Cer26:0, Cer16:1, Cer18:1, Cer20:1, and Cer22:1	Frontal cortex	Bae et al. 2014
	Voluntary alcohol drinking	Male and female mice	↓ SM 18:1 18:0, SM 18:1 18:1, and SM 18:1 20:0	Nucleus accumbens and dorsal hippocampus	Müller et al. 2017
Amphetamine	Self‐administration	Male rats	↑ total Cer ↑ Cer d18:1/18:0 and d18:1/24:0	Frontal cortex, dorsal and ventral striatum	Astarita et al. 2015
			↑ Cer d18:1/16:0	Dorsal striatum	

Abbreviations: Cer, ceramide; SM, sphingomyelin.

Alcohol is the most widely used reward substance in Western societies. Multiple clinical and preclinical data indicate the role of SLs in alcohol use, abuse, and addiction. Non‐addicted individuals usually consume alcohol in a non‐toxic safe dose range, particularly for their rewarding and instrumentalization properties. However, a small percentage of people make the transition from controlled alcohol use to abuse and addiction (Müller et al. 2023). Escalation of alcohol intake results in abuse, a compulsive use commonly associated with recurrent social, occupational, legal, or interpersonal adverse consequences. The next step, alcohol dependence, is characterized by a need for markedly increased amounts of alcohol to achieve intoxication or desired effect and development of withdrawal syndrome and tolerance. Although DSM‐IV distinguishes these two conditions, DSM‐V integrates them into a single disorder called alcohol use disorder (AUD) (Grant et al. 2007). All three conditions possess different mechanisms, and only controlled alcohol use can be considered a rewarding state positively affecting well‐being. However, all these conditions seem to be mediated by SLs.

Acute alcohol intoxication is associated with an increase in lysosomal ASM activity in the peripheral blood cells as well as secretory ASM in the blood serum of patients. This is followed by a decline during withdrawal (Reichel et al. 2011; Mühle et al. 2014). In line, NSM activity in early‐abstinent male and female patients diagnosed with alcohol use disorder upon hospital admission for detoxification treatment was also enhanced (Kalinichenko, Mühle, et al. 2021). Preclinical studies are in line with these data. Alcohol‐fed mice are characterized by enhanced levels of total ceramide, Sph, and sphinganine in the liver, although plasma concentration of ceramide remained unchanged and plasma levels of Sph and sphinganine decreased (Clugston et al. 2011). SL species composition is also affected by forced alcohol treatment. Zhao et al. (2011) showed that levels of SM16:0, SM18:1, and SM18:0 were decreased in the serum, but increased in the heart of alcohol‐fed mice. Cer18:0 and Cer16:0 were reduced in the liver of these rodents, but enhanced in the kidneys (Zhao et al. 2011). Carr et al. (2014) found an increase in Cer22:0 and Cer24:0 levels in the liver of mice fed with alcohol for 6 weeks, but no changes in concentrations of Cer16:0, Cer16:1, or Cer24:1 species (Carr et al. 2014). In turn, Sozio et al. (2010) observed an increase in Cer16:0, Cer18:0, and Cer24:0 levels in the liver of alcohol‐fed animals, while the concentration of these ceramides was decreased in alcohol‐fed ASM knockout mice (Sozio et al. 2010). Early adolescence or prenatal acute alcohol administration resulted in an increase in total ceramide concentration in the brain (Saito et al. 2010; Saito et al. 2007). Binge alcohol drinking was accompanied by a decrease in the levels of Cer26:0, Cer16:1, Cer18:1, Cer20:1, and Cer22:1 species in the FC of rodents (Table 4). However, acute withdrawal after binge drinking was followed by a significant increase in the concentrations of Cer16:0, Cer18:0, and Cer20:0 species. This was largely driven by an enhanced expression of CerS2 and CerS4 (Bae et al. 2014). Altogether, these data indicate that alcohol might induce changes in the SL metabolism of patients with alcohol use disorder or animals exposed to forced alcohol treatment. However, a toxic effect of alcohol in these cases can not be excluded and changes in the SL metabolism probably do not mirror a physiological response to alcohol use. Therefore, the second part of this chapter we will focus on the changes in the SL system in subjects during controlled use of psychoactive substances not associated with addiction‐like behavior.

Voluntary alcohol drinking in the two‐bottle free‐choice model significantly reduced the abundance of SM species, SM 18:1 18:0, SM 18:1 18:1, and SM 18:1 20:0, in the Nac and DH in wt mice (Müller et al. 2017; Table 4). However, the activities of ASM, NSM, AC, and NC did not change in the DH of wt mice voluntarily consuming alcohol compared to non‐drinkers (Kalinichenko, Mühle, et al. 2019).

Modulation of the SL system also has a strong influence on the reward‐associated behavior. Zoicas, Huber, et al. (2020) showed that intracranial administration of ceramide Cer16:0 to the basolateral amygdala, but not to the DH of male mice, increased consumption of 10% alcohol in a two‐bottle free‐choice paradigm. Although intracranial administration of Cer16:0 to the DH did not affect alcohol drinking behavior, alcohol normalized the Cer16:0‐induced depressive‐like phenotype in mice. Interestingly, ceramides Cer8:0 and Cer20:0 did not affect alcohol drinking phenotype when administered to the basolateral amygdala or the DH of male mice (Zoicas, Huber, et al. 2020). ASM overexpression in mice resulted in enhanced alcohol consumption, an enhanced alcohol deprivation effect in a free‐choice paradigm, and a facilitated establishment of the conditioned effects of alcohol in the CPP test. In line with the results of the study above, alcohol reduced innate depression‐like behavior in ASM overexpressing mice, but not in wt littermates. These data indicate the specific role of the SL system in the antidepressant effects of moderate voluntary alcohol consumption (Müller et al. 2017).

In turn, heterozygous ASM knockout mice exhibited reduced alcohol preference in a free‐choice drinking paradigm, although alcohol consumption was preserved. In the CPP test, reduced ASM activity prevented the establishment of an alcohol CPP, while conditioned hyperlocomotion was established in wt and heterozygous ASM knockout mice at comparable speeds. ASM knockout also prevented sensitization to an alcohol challenge in this test (Müller et al. 2017). On the contrary, analysis of drinking pattern in a mouse model of Niemann‐Pick diseases, a homozygous ASM knockout was associated with higher intake and preference of high, but not low concentrations of alcohol in a free‐choice alcohol drinking paradigm. However, this difference was overwritten by stress exposure (Kalinichenko, Mühle, et al. 2019). ASM specifically overexpressing in the forebrain determined sex‐specific differences in alcohol consumption behavior. Male mice with a forebrain‐specific ASM overexpression (ASMtg^fb^) consumed more alcohol in a free‐choice drinking paradigm compared to wt littermates, and their alcohol deprivation effect was more pronounced. Interestingly, binge alcohol drinking in a model with only an alcohol bottle available in the cage for 2 h did not differ between ASMtg^fb^ and wt mice, thus referring to the importance of the forebrain ASM for moderate recreational voluntary drinking behavior. Analysis of alcohol‐induced CPP showed that the forebrain ASM overexpression diminished alcohol‐induced place preference in males. Together with the drinking phenotype, diminished reinforcing properties of alcohol might indicate that the forebrain ASM overexpression in males is not responsible for high‐risk alcohol consumption. Probably, male ASMtg^fb^ mice are using alcohol to cope with stress and depression‐like behavior as shown before (Zoicas, Schumacher, et al. 2020; Müller et al. 2017), and thus maintain alcohol drinking at a stable level without developing of addiction‐like behavior (Müller et al. 2023). In turn, alcohol drinking behavior in a free‐choice paradigm did not differ in female mice with the forebrain ASM overexpression and wt littermates. In line, reinforcing properties of alcohol were also similar in these animals as shown in the CPP test. However, female ASMtg^fb^ mice consumed more alcohol in the binge alcohol drinking model. Therefore, the forebrain ASM does not contribute to the control of voluntary alcohol consumption and rewarding properties of alcohol in females, while it could mediate the mechanisms of alcohol use disorder in a sex‐specific way (Kalinichenko et al. 2025).

Another enzyme of the ceramide synthesis, NSM, is also shown to mediate alcohol drinking behavior in a non‐addictive state. The single nucleotide polymorphisms analysis performed on a population of 456 693 participants (56% female) with complete genotype and behavioral data from the UK Biobank revealed that 17 out of 26 haplotypes of the *SMPD3* gene coding for NSM are associated with alcohol use behavior, particularly parameters “alcohol intake frequency” and “alcohol drinker status”. It should be emphasized that the participants were not diagnosed with alcohol addiction, but corresponded to a normal population of the UK. A preclinical study with female mice indicated that the heterozygous NSM knockout is associated with reduced alcohol consumption in a two‐bottle free‐choice drinking test. However, NSM was neither required for the establishment, nor the retrieval of the conditioned reinforcing effects of alcohol in NSM knockout females. In line, two NSM inhibitors GW4869 and ES048 administered before CPP retrieval did not affect CPP expression in female mice (Kalinichenko, Mühle, et al. 2021). On the contrary, male full‐body heterozygous NSM knock out mice consumed more alcohol in a two‐bottle free‐choice drinking paradigm, while establishment of an alcohol CPP was delayed. However, the expression of a previously established alcohol CPP was not affected by NSM antagonist treatment with ES048 or GW4869 in male mice. Therefore, NSM in males is necessary to establish, but not to retrieve the conditioned reinforcing effects of alcohol (Kalinichenko et al. 2023).

Gangliosides also control alcohol consumption behavior in a non‐addictive state. Particularly, the alcohol‐preferring C57BL/6ByJ mouse strain exhibits significantly lower concentrations of the ganglioside GM1 in the serum, blood cells, and liver, but not in the cerebellum, when compared to the non‐preferring BALB/cJ strain. Oral self‐administration of alcohol within a preference testing paradigm resulted in a reduction of serum GM1 levels in alcohol‐preferring animals. In line, chronic alcohol drinking also led to a decrease in hepatic GM1 content (Saito et al. 2004). Alcohol drinking was also associated with a decrease in the total brain level of GD1a and an increase in GT1 amount in rats (Vrbaški et al. 1984). Analysis of brain area ganglioside distribution revealed an increase in the levels of GT1b in the hypothalamus and thalamus as well as GD1a in the thalamus, while GM1 concentration decreased in the hypothalamus and thalamus of rats exposed to chronic alcohol drinking (Vrbaški 1995).

Cocaine is one of the addictive drugs widely used for its high rewarding properties with about 20% probability of transition to dependence (Lopez‐Quintero et al. 2011). Forced cocaine administration in the CPP test significantly affects the brain lipidome of mice. It should be emphasized that the changes in the content of ceramides and gangliosides are species‐ and brain area‐dependent (Lin et al. 2017). Cocaine self‐administration in rats resulted in a reduced mRNA expression of genes of ceramide metabolism *Cers1*, dihydroceramide desaturases *Degs1* and *Degs2*, and *Smpd1* in the PFC of rats, as well as a reduction of *Cers4* expression in the striatum. These changes were reversed after 10 days of abstinence. In monkeys, cocaine‐induced place preference coincided with a reduction in blood ASM, but not NSM activity after CPP establishment (Frankowska et al. 2021). Forebrain ASM overexpression reduced cocaine‐induced place preference in male mice compared to wt littermates without affecting cocaine‐induced hyperlocomotion (Kalinichenko et al. 2025).

Ganglioside composition is also being affected by cocaine administration (Lin et al. 2017; Cabello et al. 1994; Leskawa et al. 1994). Valdomero et al. (2010) showed an interaction between a ganglioside GM1 and rewarding properties of cocaine. Treatment with GM1 before cocaine injection did not have any rewarding effects itself, but increased the rewarding effect of cocaine in the CPP test (Table 7). This enhancement correlated with a significant increase in the brain's cocaine level without affecting pharmacokinetic parameters, such as plasma bound/free cocaine ratio, a permeability of the blood–brain barrier for cocaine. It also did not influence the inhibitory effect of cocaine on the DA transporter, a major mediator of cocaine's addictive properties in the brain (Valdomero et al. 2010, 2015).

Amphetamines are among the most globally used illicit stimulants (UN Office on Drugs and Crime 2022). Self‐administration of d‐methamphetamine increases ceramide content in the FC, DS and VS, as well as in the peripheral organs of rats. An increase in the levels of Cer d18:1/18:0 and d18:1/24:0 was observed in the FC, DS and VS, while Cer d18:1/16:0 amount increased only in the DS. Interestingly, no changes in the ceramide species composition were found in the hippocampus or cerebellum of these rats. Observed changes in the DS of rats self‐administering d‐methamphetamine were associated with an increase in the mRNA levels encoding CerS1, CerS4 and CerS6, while the mRNA level of CerS2 significantly declined. Similar, mRNA levels of CerS4 and CerS6 decreased, and of CerS2 increased in the FC of rats exposed to amphetamine compared to yoked controls. A significant increase in the mRNA level of CerS6 was only found in the cerebellum of these rats (Astarita et al. 2015). It should be emphasized that these enzymes catalyze the de novo pathway of the ceramide metabolism. The sphingomyelinase pathway is also involved in the rewarding properties of amphetamine. Forebrain ASM overexpression increased amphetamine‐induced CPP in female mice compared to wt littermates without affecting cocaine‐induced hyperlocomotion. The rewarding properties of amphetamine were intact in female mice with forebrain‐specific ASM overexpression as shown in the CPP test (Kalinichenko et al. 2025).

Altogether, the SL system contributes to reward‐directed behavior in a substance‐, species‐, and sex‐specific manner. Evidence suggests that ceramides and gangliosides may directly influence or indirectly enhance goal‐directed behavior and may further drive drug consumption. However, the precise mechanisms by which these lipids integrate into the neurobiological processes underlying drug use and addiction have yet to be fully elucidated.

### 3.5 Sphingolipids in Executive Function Regulation

Attention Deficit Hyperactivity Disorder (ADHD) is a neurodevelopmental condition that typically begins in childhood and often continues into adulthood. It is characterized by age‐inappropriate attention deficits, hyperactivity, and impulsivity, affecting brain regions involved in cognitive, sensory, and motor functions. ADHD affects the individual's emotional well‐being and is associated with higher anxiety, impaired sleep, lower self‐esteem, poorer psychosocial health, and poorer overall quality of life (Travell and Visser 2006; Schatz and Rostain 2006; Danckaerts et al. 2010). Importantly, similar symptoms were observed in siblings and other family members of ADHD patients (Peasgood et al. 2016).

A small study on children with ADHD revealed lower serum levels of SM16:0, SM18:0, SM18:1, and SM24:1 as well as Cer24:0 and deoxyceramide Cer24:1 (Table 5). Notably, the low levels of Cer24:0 and deoxyceramide Cer24:1 in ADHD patients were not paralleled by a decrease in dhCer, thus suggesting no changes in CerS 2 activity and no potential involvement of the de novo pathway. Serum levels of S1P and sphinganine‐1‐phosphate did not differ between patients with ADHD compared to unaffected relatives or unaffected controls without a family history of ADHD. The observed changes in the serum SL profile may reflect delayed brain maturation and could serve as a potential biomarker to distinguish ADHD patients from unaffected individuals (Henríquez‐Henríquez et al. 2015). Interestingly, compared to these mass spectrometry data, an ELISA‐based study in unmedicated children with ADHD found total plasma ceramide and sphingomyelin, but not galactosylceramidase levels to be significantly elevated relative to neurotypical controls. Thereby, ceramide levels positively correlated with the severity of soft neurological signs, such as gait, station, and dysrhythmia scores (Güleç et al. 2025).

**TABLE 5:** Sphingolipids in executive function regulation: Changes in tissue levels of ceramides and sphingomyelins in preclinical and clinical studies.

Preclinical studies			
Model of ADHD	Changes	Tissue	References
Wistar‐Kyoto male rats	↑ total Cer ↑ Cer16:0, Cer18:0, and Cer24:1	Arterial tissue	Spijkers et al. 2011
	↑ total Cer ↑ Cer16:0, Cer22:0, Cer24:1, and Cer24:0	Plasma	
Spontaneously hypertensive male rats	↑ sphinganine, Cer d18:1/12:0 and sphingosylphosphorylcholine d18:1	Serum	Jiang et al. 2015

Clinical studies			
Patients	Changes	Tissue	References
Children with ADHD	↓ SM16:0, SM18:0, SM18:1, SM24:1 ↓ Cer24:0 and Cer24:1	Serum	Henríquez‐Henríquez et al. 2015
	↑ total Cer and SM	Plasma	Güleç et al. 2025
Adult patients with ADHD	↑ S1P d18:1 and S1P d18:0	Plasma	Brunkhorst‐Kanaan et al. 2021
Adult patients with comorbid ADHD and MDD or BP	↑ S1P d18:1 and Cer22:0	Plasma	Brunkhorst‐Kanaan et al. 2021

Abbreviations: ADHD, attention deficit hyperactivity disorder; BP, bipolar disorder; Cer, ceramide; MDD, major depressive disorder; S1P, sphingosin‐1‐phosphat; SM, sphingomyelin.

Alterations in SL metabolism have also been observed in adults with ADHD. Adult patients with ADHD were characterized by increased plasma concentrations of S1P d18:1 and S1P d18:0, but not other ceramides. In turn, patients with ADHD and comorbid major depression or bipolar disorder displayed increased S1P d18:1 as well as increased Cer22:0. Therefore, plasma SL profiles differ between patients suffering from ADHD and affective disorders. The S1P d18:1 to Cer22:0 ratio may constitute a diagnostic or prognostic tool (Brunkhorst‐Kanaan et al. 2021). In line, genetic analyses also implicated SL metabolism in ADHD. Whole‐exome sequencing of 1520 subjects revealed significant associations between ADHD and variants in eight genes encoding enzymes of the SL metabolism: *GALC*, *CERS6*, *SMPD1*, *SMPDL3B*, *CERS2*, *FADS3*, *ELOVL5*, *and CERK*. Moreover, significant associations between ADHD and variants rs4668077 and rs139609178 in the genes encoding for CERS6 and CERS2, respectively, were observed. The *SMPD1* marker rs35785620, coding for ASM, *SMPDL3B* marker rs143078230, coding for sphingomyelin phosphodiesterase, as well as *GALC* markers rs398607 and rs1805078, coding for galactosylceramidase, were associated with ADHD. Changes in these genes might not only indicate SL imbalance or myelinization deficiency typical for the disorder but also serve as pathogenetic mechanisms or diagnostic markers of ADHD and associated with it affective symptoms (Henriquez‐Henriquez et al. 2020).

Preclinical studies confirm the involvement of the SL system in ADHD. For example, mice with inactive CerS 6 gene (CerS6KO) exhibited significant hyperactivity in the open field compared to wt controls (Ebel et al. 2013). An animal model of ADHD, the spontaneously hypertensive rats, which are characterized by hyperactivity, impulsivity, and deficits in sustained attention compared with normotensive Wistar–Kyoto rats (Russell et al. 2005), showed significantly increased levels of total ceramide, but not total SM, Sph, and S1P in arterial tissue. The significant increase in total ceramide was mainly due to increased Cer16:0, Cer18:0, and Cer24:1 (Table 5). In the plasma, enhanced total ceramide levels were determined by a rise in Cer16:0, Cer22:0, Cer24:1, and Cer24:0 concentrations (Spijkers et al. 2011). In line, spontaneously hypertensive rats possessed higher serum levels of sphinganine, Cer d18:1/12:0, and sphingosylphosphorylcholine d18:1 (Jiang et al. 2015). Altogether, current evidence from human and animal studies indicates that ADHD and its influence on social interactions and well‐being are accompanied by distinct alterations in SL pathways.

### 3.6 Sphingolipids in Sleep Behavior

Sleep is a fundamental behavioral and physiological state critical for restoration and brain function. Although sleep is not considered a component of well‐being, poor sleep or insomnia has a considerable negative impact on it, particularly in reducing happiness levels (Zhao et al. 2019). Sleep quality in healthy middle‐aged American adults assessed by the Pittsburgh Sleep Quality Index significantly correlates with blood ceramide levels (Berkowitz et al. 2021). Metabolite‐Gene network analysis performed on a healthy mixed cohort of volunteers showed pronounced changes in the SL metabolic process. Particularly, the SLs Cer40:2, Cer d41:2, SM43:2, SM d33:2 were altered during insufficient sleep (Depner et al. 2020; Table 6). Sleep disturbances in patients with schizophrenia negatively correlated with a decrease in the plasma levels of Cer d18:1/22:0, Cer d18:1/24:0, and SM24:0 (Yan et al. 2024). In patients with PTSD and associated sleep disturbances, total blood levels of ceramides and SMs were altered. These changes were observed in male, but not female patients. Importantly, sleep quality and Pittsburgh Sleep Quality Index correlated with practically all studied ceramide and SM species. SMs and ceramides measured in the blood of men with PTSD were positively correlated with poorer sleep quality, while ceramides negatively correlated with total sleep time (Bhargava et al. 2024). SLs might also serve as markers of insomnia. Military personnel suffering from insomnia and successfully exposed to 3 months of sleep‐focused cognitive behavioral treatment, which improved insomnia symptoms, were characterized by significantly enhanced expression of the *SGMS2* gene, coding for SMS2 (Livingston et al. 2015). Sleep deprivation in healthy male volunteers was associated with an increase in three SMs, SM14:1, SM20:2 and SM22:2 (Davies et al. 2014). In line, the blood level of SM18:1/24:1 was also associated with sleep deprivation (Chua et al. 2015). Integrative metabolomic analysis indicated a role of SL metabolic pathways in mediating the effects of sleep deprivation on memory (Chen et al. 2025). In line, enrichment analysis performed in *Caenorhabditis elegans* with a reduced sleep phenotype revealed SL metabolism as a key pathway that controls sleep. The strongest enrichment among all the determined genes of interest was observed for several SL metabolism genes, hyl‐1, hyl‐2, F33D4.4, sms‐3, hpo‐13, sptl‐3, gba‐1, and plpp‐1.2. Metabolome analysis showed that aptf‐1 knockout mutants characterized by a virtually complete lack of sleep during L1 arrest display a significant depletion in the levels of multiple SMs, including SM d18:0/16:1, SM d18:1/26:1 / SM d18:0/26:1, SM d18:0/18:0, SM d18:0/12:0, SM d18:0/22:3, SM d18:0/14:0, SM d18:0/22:1(OH), SM d17:1/24:0, SM d19:1/24:1, SM d18:1/18:1/SM d18:1/18:1, SM d18:1/22:1, SM d18:1/22:0/SM d18:0/22:1, SM d18:0/26:0, and SM d18:1/24:1. However, sleep loss in these subjects was accompanied by an increase in the levels of SM d18:1/26:0 and SM d18:1/12:0. The authors emphasized that functional SL metabolism is critical for normal sleep behavior and energy conservation during developmental arrest (Koutsoumparis et al. 2023).

**TABLE 6:** Sphingolipids in sleep behavior: Changes in tissue levels of ceramides and sphingomyelins in clinical studies.

Medical condition associated with sleep loss	Gender	Changes	Tissue	References
Insufficient sleep	Males	Altered Cer40:2, Cer d41:2, SM43:2, SM d33:2	Plasma	Depner et al. 2020
Schizophrenia	Males and females	↓ Cer d18:1/22:0, Cer d18:1/24:0, and SM24:0	Blood	Yan et al. 2024
Sleep deprivation	Males	↑ SM14:1, SM20:2 and SM22:2	Plasma	Davies et al. 2014
	Males	↑ SM18:1/24:1	Plasma	Chua et al. 2015

Abbreviations: Cer, ceramide; SM, sphingomyelin.

**TABLE 7:** Effects of interventions in ganglioside metabolism on various behavioral patterns of emotional well‐being in rodents.

Ganglioside	Type of intervention	Behavioral effect	References
GM1	GM1 administration	↓ depression‐ and anxiety‐like behavior ↓ anxiety‐like behavior ↓ anhedonia	Alpaugh et al. 2017 Yin et al. 2023 Jiang et al. 2016
		↓ social avoidance ↑ social interaction	Jiang et al. 2016 Yin et al. 2023
		Reverses impaired memory in the Morris water maze test	Yin et al. 2023
		↑ rewarding effects of cocaine	Valdomero et al. 2010
GD3	GD3 synthase knockout mice	↑ depression‐like behavior	Wang et al. 2014
		↓ memory in the Barnes maze and in the novel object recognition tests	Tang et al. 2021
GM2	GM2/GD2 synthase knockout mice	↓ memory in the Morris water maze test and step‐down inhibitory avoidance task	Sha et al. 2014
	Double GM2/GD2 synthase and GD3 synthase knockout in mice	↓ memory in the 8‐arm radial maze	Tajima et al. 2010

Gangliosides were also shown to participate in sleep regulation. A study of Islam et al. (2002) showed that selenium in the dose of 0.2 mg/kg, which inhibits sleep in rats, induced a decrease in the total ganglioside level in two sleep‐promoting regions of the brain, the preoptic area and brain stem. However, an increase in ganglioside amount was observed in the wakefulness‐promoting posterior hypothalamus (Islam et al. 2002). Glucocerebrosidase (GBA) is a lysosomal enzyme responsible for breaking down the glycosphingolipid glucosylceramide (GluCer) into ceramide and glucose. Two genetic studies indicated a strong association between GBA variants and idiopathic rapid eye movement sleep behavior disorder (iRBD) (Gan‐Or et al. 2015; Krohn et al. 2020). Among individuals with Parkinson's disease, GBA mutation carriers were also more likely to report RBD symptoms (Gan‐Or et al. 2015). Moreover, carriers of several GBA variants exhibited earlier onset of RBD and a faster progression to overt neurodegeneration, highlighting GBA's role not only in disease risk, but in sleep‐related neurodegenerative trajectories (Krohn et al. 2020). A comprehensive study by Vaughen et al. (2022) demonstrated that glial expression of GBA (gba1b) in Drosophila is essential for circadian remodeling and sleep regulation. Their work revealed that specific glial cells degrade SLs via Gba1b, enabling diurnal cycling of brain SLs, including GluCer and ceramides. In gba1b mutants, these lipids accumulated abnormally, particularly three GluCer and ceramide species with identical hydrocarbon chains: 14:1/18:0, 14:1/20:0, and 14:1/22:0, leading to disrupted sleep behavior. Moreover, circadian fluctuations of ceramide and GluCer with carbon chains 14:1/18:0 and 14:1/20:1, as well as ceramide phosphoethanolamine 14:1/18:0 and 14:2/20:0, and GluCer 15:0/22:1 and 14:0/16:0 were found in Drosophila. In *gba1b* ^*Δ*^ mutants, GluCer and ceramide levels are elevated and fail to cycle, which was accompanied by deficits in activity and sleep. The authors propose that both SL biosynthesis and degradation are crucial for the diurnal remodeling of circadian clock neurites. Therefore, the observed circadian SL changes might enable diurnal circuit remodeling and proper circadian behavior (Vaughen et al. 2022). Altogether, the present findings might uncover a principally new field of SL‐based sleep disorders, particularly sleep impairments comorbid with other psychiatric conditions, such as depression, where the role of SLs is already confirmed.

## 4 Potential Neurobiological Mechanisms of Contribution of Sphingolipids to Emotional Well‐Being

### 4.1 Role of Sphingolipids in Regulation of Neuronal Death and Neurogenesis

**FIGURE 1:**
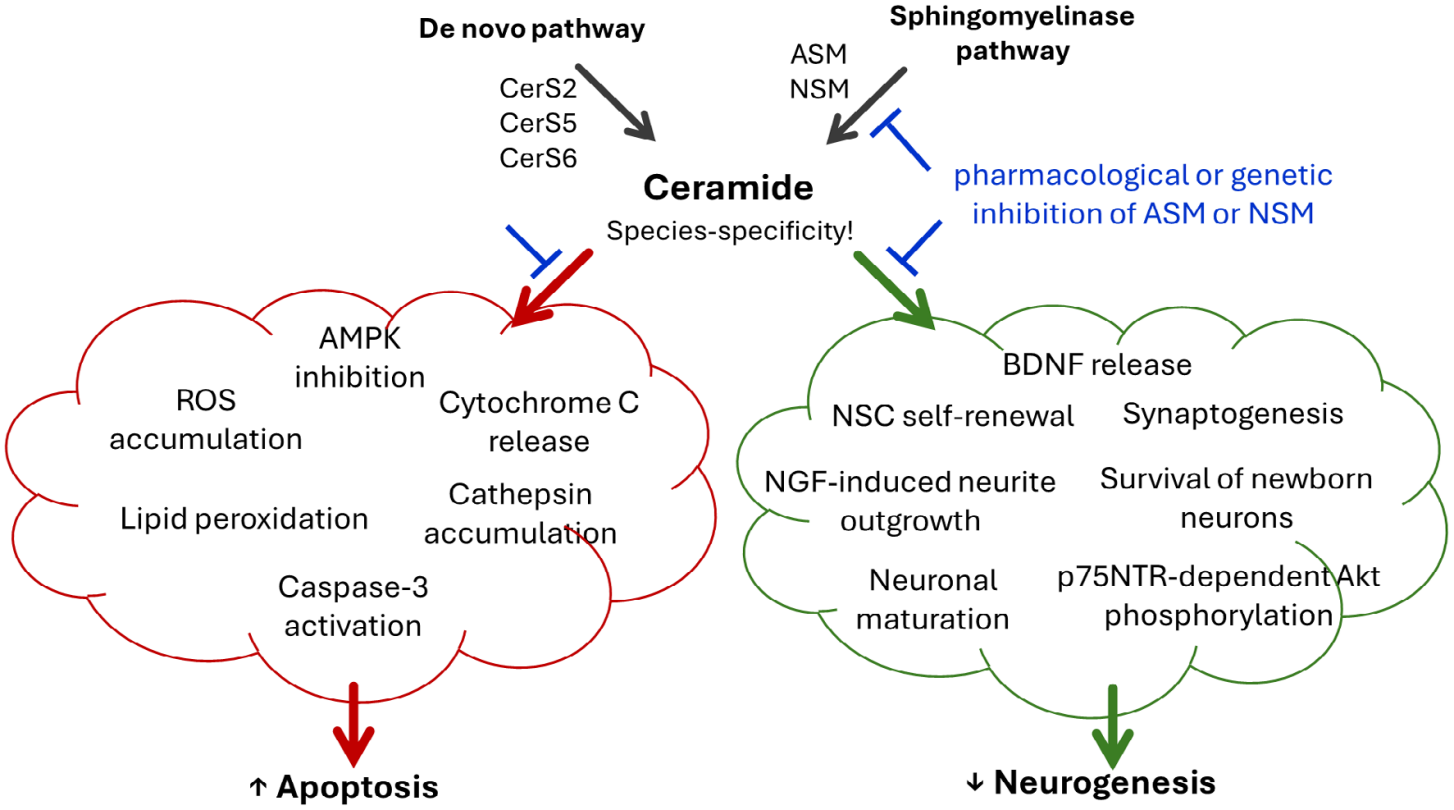
Sphingolipid regulation of neuronal death and neurogenesis in the brain. Sphingolipids regulate the balance between neuronal death and neurogenesis through various cellular mechanisms. Enzymes of sphingolipid metabolism act as critical molecular switches, with context‐, species‐, and sex‐specific effects. AMPK, α‐amino‐3‐hydroxy‐5‐methyl‐4‐isoxazolepropionic acid; ASM, acid sphingomyelinase; BDNF, brain‐derived neurotrophic factor; CerS, ceramide synthase; NGF, nerve growth factor; NSC, neural stem cell; NSM, neutral sphingomyelinase; p75NTR, p75 neurotrophin receptor; ROS, reactive oxygen species.

The ratio between neuronal death and neurogenesis in the adult brain is one of the key mechanisms determining optimal brain functioning. This ratio is often impaired during mental disorders, such as cognitive deficits, affective states and dysfunctional substance use (Sapolsky 2000; Duman and Monteggia 2006; Parul et al. 2021; Ballesta et al. 2019). Ceramides have been shown to modulate both facets of the apoptotic/neurogenic balance. Ceramides, and particularly the ratio between ceramide and its metabolite Sph, determine initiation and progression of apoptosis across various cell types (Cuvillier et al. 1996; Hoekstra 1999; Tsugane et al. 1999). Ceramides facilitate oxidative stress and pro‐apoptotic signaling, leading to accumulation of reactive oxygen species (ROS), increased lipid peroxidation, inhibition of AMP‐activated protein kinase (AMPK), release of cytochrome c, accumulation of cathepsins, and ultimately, cell death (Anderson and Borlak 2008; Deaciuc et al. 2000; Liangpunsakul et al. 2010; Heinrich et al. 1999; García‐Ruiz et al. 1997). In turn, ROS and pro‐inflammatory mediators may upregulate ceramide synthesis (Lim et al. 2011). Enzymes involved in ceramide biosynthesis, such as ASM, have been shown to facilitate endoplasmic reticulum stress followed by cell apoptosis (García‐Ruiz et al. 1997). This process can be attenuated by pharmacological or genetic inhibition of ASM (Fernandez et al. 2013; Yang et al. 2016). In vivo studies also indicate that apoptotic neurodegeneration in the cortex, hippocampus, and inferior colliculus of 7‐day‐old mice exposed to alcohol was associated with elevated ceramide levels and caspase‐3 activation in the forebrain (Saito et al. 2010). Clinical studies further implicate the ceramide system as a mediator of alcohol‐induced apoptosis. Neurodegeneration, white matter atrophy, and elevated oxidative stress in individuals with chronic alcohol use are associated with increased expression of genes involved in ceramide biosynthesis (De la Monte et al. 2009). It should be emphasized that there is no consensus if all ceramide species are mediating apoptosis to the same extent. For example, Cer16 was shown to be anti‐apoptotic and Cer18 as pro‐apoptotic in cells derived from squamous cell carcinoma (Senkal et al. 2011). On the contrary, Cer16 in Hela cells was inducing apoptosis under various conditions (Mesicek et al. 2010; Sassa et al. 2012). Very‐long‐chain, but not long‐chain ceramides induce cell death in cardiomyocytes (Law et al. 2018). The effects of ceramides on the apoptosis are shown to be mediated by the enzymes of the de novo pathway of synthesis, particularly CerS2, 5, and 6 (Mesicek et al. 2010; Senkal et al. 2011), as well as enzymes of the sphingomyelinase pathway, NSM and ASM (Yabu et al. 2015; García‐Ruiz et al. 1997). These findings underscore the specific role of the ceramide system in regulating cell death, potentially accounting for the observed deleterious effects of elevated ceramide levels on emotional behavior (Figure 1).

Conversely, ceramides also impact neurogenesis in the adult brain. Gulbins et al. (2013) demonstrated that transgenic mice overexpressing ASM or AC exhibit deficits in neurogenesis, neuronal maturation, and neuronal survival. Interestingly, antidepressants such as amitriptyline and fluoxetine, which function as FIASMAs, restored neurogenesis in the hippocampus. These effects were not observed in ASM knockout mice (Gulbins et al. 2015, 2013). Pharmacological inhibition of ASM also reversed impaired neuronal regeneration in mice with mild traumatic brain injury (Niziolek et al. 2021) or neurogenesis in rats suppressed by reserpine administration (Yang, Yu, et al. 2020). Likewise, the neurogenic and antidepressant effects of melatonin in stressed mice are mediated via ASM inhibition. The beneficial effects of melatonin were not observed in ASM knockout mice (Hoehn et al. 2016). In line, a reduction of NSM activity in male mice resulted in an increase in neural stem and early progenitor cells, but did not affect the number of proliferating or activated stem cells or the number of newborn neurons and neuroblasts. In female mice, NSM hypoexpression was shown not to affect neurogenesis (Kalinichenko et al. 2023; Kalinichenko, Mühle, et al. 2021). Taken together, these findings suggest that the ceramide system acts as a negative regulator of neurogenesis, neuronal maturation, and survival, probably in a sex‐specific way.

One of the mechanisms of the effects of the SLs on neurogenesis might be dependent on the brain neurotrophic system. It was shown that signaling from the key neurotrophin receptors, tyrosine kinase receptor B (TrkB) and the p75 neurotrophin receptor (p75NTR), occurs within ceramide‐enriched microdomains of membranes (Guirland and Zheng 2007). In vitro studies showed that Cer8:0 can directly promote brain‐derived neurotrophic factor (BDNF) release from microglial cells mediated by protein kinase C delta (PKCδ) and/or epsilon (PKCε) without affecting the secretion of TNF‐α, interleukin‐1β, or nitric oxide (Nakajima et al. 2002). Inhibition of NSM, either by a specific inhibitor or siRNA‐mediated knockdown, abolished the neuroprotective effects of BDNF (Candalija et al. 2014). In addition, NSM regulates NGF‐induced neurite outgrowth and synaptogenesis (Brann et al. 1999; Hirata et al. 2001; Ito and Horigome 2002). A specific interaction between p75NTR and ASM has also been described. Roux et al. (2001) found that p75NTR‐dependent Akt phosphorylation, a critical mechanism of Trk‐mediated cell survival, is diminished in fibroblasts from patients with Niemann–Pick disease deficient in ASM, but not NSM (Roux et al. 2001). These findings suggest that ceramides, along with the enzymes involved in their metabolism, play a regulatory role in modulating neurotrophin signaling.

Gangliosides are also shown to affect neuronal growth. In particular, long‐term intraperitoneal GM1 administration restored cell proliferation, promoted long‐term survival and neuronal differentiation in the DH of mice with brain injury (Zhang et al. 2005). Lack of GD3, one of the predominant gangliosides expressed in neural stem cells (NSCs), in mice deficient in sialyltransferase (ST) II and III, the key enzymes regulating biosynthesis of c‐series gangliosides, was associated with a marked reduction in NSC self‐renewal capacity (Itokazu et al. 2024). A depletion of NSCs in the dentate gyrus and subventricular zone of adult ST‐II knockout mice resulted in a reduction in the volume of these regions and development of a depression‐like behavioral phenotype (Wang et al. 2014). Ganglioside GD3 and GM1 increased the number of doublecortin‐expressing immature neurons in the olfactory bulb. Nasal GD3 administration rescued the neuronal populations in the periglomerular layer of A53T alpha‐synuclein‐expressing mice, a model of early onset Parkinson's disorder (Fuchigami et al. 2023). Protective properties of gangliosides might be mediated by their strong interaction with neurotropic factors. The ganglioside GM1 affects the release of BDNF, neurotrophin‐3, and nerve growth factor in various cell types (Rabin et al. 2002; Lim et al. 2011). GT1b and GQ1b may also promote in vitro BDNF secretion, potentially via an NMDA receptor signaling pathway (Lim et al. 2011). In vivo studies are in line with the in vitro data. An intracerebroventricular injection of GQ1b significantly enhanced BDNF levels in the PFC and hippocampus of rats (Shin et al. 2014). One of the mechanisms of ganglioside effects on neurotrophin signaling might be mediated by the interaction between specific neurotrophin receptors, particularly of the Trk receptor family, and gangliosides of lipid‐enriched domains in membranes (Ferrari et al. 1995; Duchemin et al. 2002, 2008; Rabin and Mocchetti 1995; Rabin et al. 2002). Cells lacking GM1 or exposed to anti‐GM1 antibodies also lack TrkA receptors or abolish Trk activation, which leads to a failure in signal transmission (Mutoh et al. 2002; Ueda et al. 2010).

The interaction between gangliosides and neurotrophic factors may be a key determinant of their effects on neuronal growth, dendritogenesis, and synaptogenesis. GD3, in particular, promotes the proliferation of NSCs, thereby increasing the number of newly generated neurons. This ganglioside supports the maturation of nascent neurons by enhancing dendritic outgrowth and branching, while a reduced density of dendritic spines is observed in neurons from GD3 synthase knockout mice. Notably, the impairments in self‐renewal capacity and radial glia‐like stem cell outgrowth in postnatal GD3S knockout NSCs were fully rescued by restoring GD3 expression (Wang et al. 2014).

Altogether, the ratio between apoptosis and neurogenesis as well as neuronal growth and branching was shown to be affected by the members of the SL family. However, the species specificity of these processes is still of specific interest.

### 4.2 Role of Sphingolipids in Regulation of Neurotransmitter Systems and Synaptic Transmission

Another mechanism by which lipids might influence the emotional state of an organism involves the modulation of neurotransmitter systems in the brain. Neurotransmitters such as 5‐HT, DA, glutamate, acetylcholine (ACh) and NA constitute a major component of the modulatory input to higher brain regions, thereby contributing to cognitive functions, emotional state and reward. The mechanisms underlying interactions between monoamines and the SL system might be determined by the presence of ceramide‐enriched domain composition. As these domains are especially enriched in receptors, and particularly G‐protein coupled receptors, changes in their organization affect receptor clustering and conformation. These processes may alter receptor function and intracellular signaling cascades (Fallahi‐Sichani and Linderman 2009). The composition of ceramide‐enriched domains is determined by ceramide‐metabolizing enzymes, which are shown to affect neurotransmitter release and turnover. Particularly, Müller et al. (2017) reported significantly reduced levels of 5‐HT and slightly diminished DA concentration in the PFC, DH, and VS of transgenic mice overexpressing ASM, coinciding with depression‐like behavior (Müller et al. 2017). Basal extracellular monoamine levels were also altered in ASM overexpressing mice: while extracellular DA concentration in the Nac and DH was reduced, 5‐HT and NA levels were unaffected. ASM overexpression also modified monoaminergic responses to stimulation: DA responses to alcohol were potentiated in these brain structures, whereas NA responses were attenuated. Interestingly, DA and NA responses to food stimuli remained unchanged, but the 5‐HT response in the Nac was enhanced (Kalinichenko, Hammad, et al. 2019). An immunohistochemical analysis of the DH showed a preserved dopaminergic and serotonergic innervation in ASM overexpressing mice (Kalinichenko, Hammad, et al. 2019). Another study in naïve Wistar rats revealed negative associations between ASM activity and monoamine concentrations. Specifically, ASM activity negatively correlated with DA levels in the ventral mesencephalon and hypothalamus, 5‐HT in the DH, ventral mesencephalon, and hypothalamus, and NA in the ventral mesencephalon, hypothalamus, and DH. Therefore, ASM activity may exert a predominantly inhibitory influence on monoaminergic signaling (Kalinichenko, Abdel‐Hafiz, et al. 2021). NSM activity has also been implicated in dopaminergic function. Kalinichenko, Abdel‐Hafiz, et al. (2021) reported a positive correlation between NSM activity and DA levels in the DS, but not in other brain regions. In turn, no significant correlations between NSM and levels of other neurotransmitters were identified. Furthermore, NSM hypoexpression in male mice was associated with reduced basal extracellular DA levels in the DH and increased 5‐HT levels in the Nac. Response of these neurotransmitters to an alcohol injection was also altered: the DA increase in the Nac was enhanced, but the 5‐HT increase in the DH was attenuated in NSM heterozygous male mice (Kalinichenko et al. 2023). Interestingly, basal levels of these monoamines were preserved in female mice with NSM knockout, although DA response to alcohol in the Nac was enhanced (Kalinichenko, Mühle, et al. 2021). In line, NSM inhibition increased DA release in both cultured synaptosomes and PC12 cells (Jeon et al. 2005; Kim et al. 2010), implicating the sphingomyelinase pathway in the regulation of dopaminergic activity. Furthermore, in vivo inhibition of SPT by myriocin reduced brain ceramide levels and increased striatal and hippocampal DA concentrations, supporting a role for the de novo ceramide synthesis pathway in DA homeostasis (Osuchowski et al. 2004). The activity of the ceramide degrading enzyme NC has been negatively correlated with 5‐HT levels in the ventral mesencephalon, and NA levels in the hypothalamus of rats (Kalinichenko, Abdel‐Hafiz, et al. 2021).

Ceramides themselves also affect the monoaminergic turnover. In vitro studies showed that a short‐chain Cer2:0 decreased DA uptake in rat striatal synaptosomes, an effect reversed by ceramide washout (Riddle et al. 2003). In contrast, Cer6:0 enhanced DA uptake in PC12 cells, but only in the presence of a calcium ionophore (Jeon et al. 2005; Kim et al. 2010). Cer8:1‐phosphate increased DA release regardless of ionophore presence, while natural Cer6:0 did not affect DA release (Jeon et al. 2005).

Emerging literature indicates the crucial role of the ceramide system in neurotransmitter signaling. Sphingomyelinase treatment in CHO cells enhanced ligand binding to 5‐HT_1A_ receptors, likely via increased receptor affinity due to altered membrane order (Jafurulla et al. 2008). However, in native hippocampal membranes, sphingomyelinase reduced agonist binding without changing membrane order (Singh and Chattopadhyay 2012). The de novo ceramide synthesis pathway also modulates 5‐HT_1A_ receptor signaling. 5‐HT_1A_ agonist binding increased in B‐lymphocytes deficient in long‐chain base 1 of SPT, an effect which was reversed by exogenous ceramide (Jafurulla et al. 2017). Inhibition of CerS with fumonisin B1 reduced 5‐HT_1A_ receptor binding, signaling, membrane mobility, and maximum 5‐HT binding to 5‐HT_7_ receptors in CHO‐K1 cells (Sjögren and Svenningsson 2007; Ganguly et al. 2011). Forebrain ASM overexpression in female mice was associated with reduced mRNA expression of receptors 5‐HT_2A_ in the Nac and 5‐HT_2C_ in the DH, while the expression of 5‐HT_1A_ receptor in both structures was preserved. Importantly, forebrain ASM overexpression in male mice was not associated with changes in serotonergic receptor expression (Kalinichenko et al. 2025).

The ceramide system has also been shown to interact with glutamatergic receptors. Kalinichenko, Abdel‐Hafiz, et al. (2021) reported reduced expression levels of GluN2B subunits in the VS and both GluN2A and GluN2B subunits in the DS in female mice with NSM hypoexpression (Kalinichenko, Abdel‐Hafiz, et al. 2021). It is estimated that more than 50% of NMDA receptors are localized within lipid rafts (Besshoh et al. 2005; Füllekrug and Simons 2004; Hering et al. 2003). In line, Wheeler et al. (2009) demonstrated that increased ceramide concentrations are associated with elevated numbers of NMDA receptor subtypes in lipid domains at hippocampal synapses. In this study it was shown that NSM is essential for TNFα‐induced NMDA receptor trafficking and synaptic modulation, as pharmacological or genetic inhibition of NSM disrupted TNFα‐induced ceramide production and impaired the associated phosphorylation and lipid domain clustering of GluN1 receptor subunits (Wheeler et al. 2009). Another enzyme of ceramide degradation, AC, is also implicated in NMDA receptor signaling. AC inhibition by d‐NMAPPD results in an increase in NMDA receptor‐mediated field excitatory postsynaptic potentials (fEPSPs) in CA1 synapses of rat hippocampal slices and a rise in GluN2B receptor activity. Probably, ceramide accumulation induced by AC inhibition increases phosphorylation of the GluN2B subunit at tyrosine 1472, without affecting serine residues 896 and 897 of GluN1 (Laurier‐Laurin et al. 2014). Consistent with these findings, exposure of hippocampal slices to a short‐chain Cer2:0 also attenuated GluN2B phosphorylation at the Tyr1472 site, indicating that ceramide modulates GluN2B receptor activity in a residue‐specific manner (Laurier‐Laurin et al. 2014).

Complex ceramides, such as gangliosides, may directly interact with 5‐HT. The positively charged amino group of 5‐HT can engage with the negatively charged sialic acid of GM1, resulting in a low to moderate affinity for the 5‐HT/GM1 complex. Moreover, the CH3 from the *N*‐acetyl group of GM1's sialic acid points towards 5‐HT's aromatic ring, establishing a CH–Pi interaction (Nishio et al. 1995). Thus, the SL binding domain of 5‐HT_1A_ receptor binds directly to GM1, which triggers a conformational change in the tryptophan residue, moving it away from the receptor's central lumen. This direct influence of GM1 on the extracellular loop 1 of the 5‐HT_1A_ receptor could potentially affect ligand binding and the receptor's functionality (Prasanna et al. 2016). Another potential mechanism underlying the interaction between gangliosides and 5‐HT involves the destabilization of 5‐HT aggregates by these lipids. When released into the synaptic cleft, 5‐HT aggregates are electrostatically attracted to gangliosides at the postsynaptic membrane, particularly GM1. GM1 disrupts the aggregates and binds 5‐HT monomers. This process facilitates the access of 5‐HT monomers to their specific protein receptors on the postsynaptic membrane (Fantini and Barrantes 2009). In line, inhibition of ganglioside synthesis in cells stably expressing 5‐HT_1A_ receptors with PDMP, a specific inhibitor of glucosylceramide synthase activity, reduces the specific 5‐HT_1A_—and 5‐HT_7_ receptor agonists binding. This is associated with a reduction in total receptor binding sites as well as a downregulation of the 5‐HT_1A_ receptor expression level (Singh and Chattopadhyay 2012). Similarly, PDMP decreased the maximum 5‐HT binding to 5‐HT_7_ receptors, but without associated changes in 5‐HT_7_ receptor expression (Sjögren and Svenningsson 2007). Moreover, GM1 administration was shown to reduce depression‐like behavior in a model of Huntington disorder, and this effect is probably determined by an increase in the cortical 5‐HT level of and a decrease in the levels of a product of 5‐HT catabolism, 5‐hydroxyindoleacetic acid (5‐HIAA), indicating decreased 5‐HT turnover in mice treated with GM1 (Alpaugh et al. 2017).

Beneficial effects of ganglioside treatment during neuronal damages described in previous chapters could be related to their effects on the serotonergic system. Subchronic daily intraperitoneal injections of GM1 recovered 5‐HT and 5‐HIAA tissue levels in the ipsilateral hippocampus following electrolytic damage near the nucleus interpeduncularis. However, GM1 did not affect tryptophan or nerve growth factor content (Vaccarino et al. 1987; Lombardi et al. 1988). Similarly, GM1 restored 5‐HT and 5‐HIAA levels in the caudal cortex 2 weeks post‐transection of monoaminergic cortical innervation, with effects lasting for several weeks (Shigemori et al. 1990). Immediate application of GM1 post‐occlusion of the left middle cerebral artery reduced the extent of post‐ischemic damage by restoring 5‐HT neurons (Ahad et al. 2020; Koga et al. 1990). 5‐HT neuron growth and nerve terminal increase were also promoted by GM1 pre‐treatment following infusion of the neurotoxin, 6‐hydroxytryptamine (Jonsson et al. 1984; Kojima et al. 1988). All these findings suggest beneficial and restorative impacts of exogenous gangliosides on the 5‐HT system. Nonetheless, it remains unclear whether these effects are due to changes in membrane ganglioside composition or their indirect effects via other biological molecules. GM1 was also found to increase the affinity of neuronal DA transporters in the membranes of rat striatal synaptosomes (Barrier et al. 2003).

Another key neurotransmitter shown to interact with gangliosides is acetylcholine (ACh). The gangliosides GM1 and GQ1b enhance depolarisation‐induced ACh release from synaptosomes (Ando et al. 1998) and facilitate tetanus‐induced long‐term potentiation in hippocampal CA1 neurons in rats (Furuse et al. 1998). GM1 has been reported to increase ACh content, choline acetyltransferase activity, and high‐affinity choline uptake in the striatum, as well as enhance choline uptake in the hippocampus of aged animals (Fong et al. 1995; Hadjiconstantinou et al. 1992). Reduction of choline uptake induced by intracerebral vincristine injections could be prevented by GM1 administration (Di Patre et al. 1989). Incubation of cortical synaptosomes with GM1 also counteracted the reduction in choline uptake caused by the choline reuptake inhibitor 3,α,α‐bis[di(2‐chloroethyl)amino]‐4,4′‐2‐biacetophenone (Maysinger et al. 1992). Furthermore, Mahadik and Mukherjee (1995) observed that GM1 co‐treatment potentiated the haloperidol‐induced increase in choline acetyltransferase activity in the striatum, hippocampus, and cerebral cortex (Mahadik and Mukherjee 1995).

In summary, substantial evidence supports both direct and indirect interactions between the SL and brain neurotransmission systems emphasizing the key role of membranal SLs in neurotransmitter signaling (Kalinichenko et al. 2024). These interactions likely contribute to the role of SLs in normal brain functioning necessary for emotional well‐being, as well as in neuropsychiatric conditions such as depression, anxiety, cognitive deficiencies, and drug dependence.

### 4.3 Role of Sphingolipids in Regulation of Neural Activity and Plasticity

SLs are the key compounds of biological membranes, particularly in the synaptic clefts. As described above, SLs compose lipid‐enriched domains in membranes, whose organization is being changed by various stimuli in a very rapid manner (Nichols et al. 2001). Considering these lipid domains as a crucial unit for extra‐ and intracellular signaling in neurons, it is proposed that SLs significantly contribute to neural activity and plasticity. Particularly, inhibition of ceramide synthesis by the specific ASM inhibitor ARC39 enhanced excitatory synaptic input onto ventral hippocampal CA1 pyramidal cells. ASM inhibition, which most likely contributed to secretory ASM, reduced firing and membrane hyperpolarization in the CA1 pyramidal cells. ARC39 induced a significant increase in the frequency and amplitude of spontaneous and miniature IPSCs, thereby contributing to the control of pyramidal cell excitability. This effect was largely reversed by the blockage of GABAA receptors. ARC39 increased the frequency of both mIPSCs and mEPSCs. Thus, ASM might act at inhibitory and excitatory transmission sites, respectively. The interaction between ASM and GABA was further confirmed by the drug's ability to enhance the inhibitory synaptic drive onto pyramidal cells. If the synaptic input of the pyramidal cells was pharmacologically isolated, the overall effect of ARC39 on cell firing was inhibitory, although some neurons displayed a biphasic response with a transient increase in firing. These data suggest that ASM inhibition has cell‐specific effects in firing properties. Similar effects were observed in ASM knockout mice. spEPSCs from ASM knockout neurons occurred at a significantly higher frequency and had a significantly larger amplitude. It should be emphasized that ARC39 failed to affect glutamatergic and GABAergic synapses in the hippocampus of ASM knockout mice (Lin et al. 2021).

The effects of the SL system on neuronal activity were also shown on the systemic level. Female mice with NSM hypoexpression had a bigger volume of DH and insular cortex. An increase in PSD thickness observed in these mice may contribute to the overall increase in hippocampal volume. This was consistent with an association of an *Smpd3* haplotype and increased hippocampal volume in humans (Kalinichenko, Mühle, et al. 2021). Resting state fMRI analysis of the adult brain connectome also revealed largely enhanced functional connectivity of the somatosensory, motor, and association cortices and the amygdala of mice with NSM hypoexpression compared to wt mice. This might explain their advantageous affective state, as they are characterized by reduced depression and anxiety‐like behavior (Kalinichenko, Mühle, et al. 2021). Analysis of brain responsiveness using fMRI showed that female mice with NSM hypoexpression react more sensitively to an acute alcohol administration, as their brain response as measured by resting state functional connectivity started earlier and lasted longer than that of wt mice. However, NSM hypoexpression was associated with a weaker regional cerebral blood volume response to acute alcohol administration. Free‐choice chronic alcohol consumption significantly increased total resting state functional connectivity in the somatosensory, motor, association, and paralimbic cortices, thalamus, hypothalamus, amygdala, hippocampus, cerebellum, and brainstem in mice with NSM hypoexpression, but not wt littermates (Wank et al. 2024). As distinct from females, male mice with NSM hypoexpression had lower volumes of DH, VS, and cerebellum. These changes were not associated with enhanced oxidative stress or diminished neurogenesis (Kalinichenko et al. 2023).

Altogether, these data indicate that the contribution of SL system to brain functioning is not limited by its structural function. It seems that the members of this system may directly affect brain plasticity at different levels and via different mechanisms. It should be noted that other mechanisms of SL effects on emotional health are still possible. Contribution of SLs into inflammatory process (Nixon 2009; Lee et al. 2023), hormonal balance (Lucki and Sewer 2008), different types of signal transduction in cells (Merrill et al. 2001), and other mechanisms might also play an important role in the discussed behavioral processes and, at the end, emotional well‐being.

## 5 Sphingolipids as Prognostic Markers in Well‐Being

Although in this review we are focusing on a healthy state determining an individual's well‐being, it should be noted that disturbances in SL metabolism are widely shown as a pathogenetic mechanism of peripheral and central disorders. Recent genetic association studies indicate that variations in SL genes are linked to numerous systemic conditions and diseases (Ottensmann et al. 2023). Among them are insulin resistance and type II diabetes (Roeske‐Nielsen et al. 2009), hypertension (Fenger et al. 2015), coronary artery disease (Ottensmann et al. 2023), metabolic syndrome (Lee et al. 2022), liver disorders including nonalcoholic fatty liver disease, alcoholic liver disease, liver cirrhosis, hepatocellular carcinoma (Ottensmann et al. 2023) and others. Moreover, deviations in behavioral representations of well‐being, which result in the development of neuropsychological disorders, are also associated with SL species. For example, a recent study based on the data from UK Biobank showed that the SL metabolism pathway is less enriched with rare disease‐causing variants in patients with major depressive disorder, indicating that this gene set contributes to the pathophysiology of this mental disorder (Zhou et al. 2021). Associations between depression and SL metabolism were also shown by Kalinichenko, Abdel‐Hafiz, et al. (2021). In an association study on healthy participants from the UK Biobank, the *SMPD3* gene coding for NSM was shown to be linked to behavioral outcomes including alcohol consumption, as well as to anxiety and depressive symptoms. 18 haplotypes of this gene showed significant associations with at least one behavioral measure, with the alcohol variables having the most significant univariate associations (Kalinichenko, Mühle, et al. 2021).

A gene‐based association analysis in 1576 participants of the Alzheimer's disease neuroimaging initiative (ADNI) identified genetic variants linked to 7 SL genes (*CERS2*, *CERS3*, *CERS6*, *ACER2*, *PLPP2*, *SPHK2*, and *DEGS1*) to be significantly associated with Alzheimer's disorder and its biomarkers, which covered the whole spectrum of Amyloid, Tau, Neurodegeneration, Cognition (A‐T‐N‐C) measures. Variations in another gene, *SPTLC3*, were associated with cognitive performance, brain atrophy in focal regions of the bilateral temporal and frontal lobes and FDG‐PET measures in the bilateral temporal and parietal lobes of patients with Alzheimer's disease. Associations between *SGMS1* and brain glucose metabolism measured by region of interest‐based FDG‐PET as well as cortical thickness in the bilateral temporal, parietal, and frontal lobes were also observed (Baloni et al. 2022). Another genome‐wide association meta‐analysis on Alzheimer's disease‐associated dementia yielded an association with the *SMPD2* gene (Beecham et al. 2014).

ADHD is another health condition associated with disrupted well‐being and mediated by dysregulated SL metabolism. A meta‐analysis using SNPs observed significant associations between ADHD and variants in eight genes determining SL synthesis (*GALC*, *CERS6*, *SMPD1*, *SMPDL3B*, *CERS2*, *FADS3*, *ELOVL5*, and *CERK*) (Henriquez‐Henriquez et al. 2020). A GWAS study focused on patients with schizophrenia revealed that three SNPs in SMPD3, coding for NSM, showed a significant association with this disorder, while no associations were found for *SMPD1*, *ASAH1*, and *ASAH2*. This study also observed significant expression alterations for *SMPD1*, coding for ASM, in the prefrontal cortex, but not in the associative striatum or hippocampus of schizophrenia patients from the Gene Expression Omnibus (GEO) database (Chestnykh et al. 2025). Another study focused on the causal associations between circulating plasma metabolites and autism. It used large‐scale GWAS datasets and showed strong negative associations between the circulating level of SM d18:1/20:1 and ASD and positive associations between the circulating level of SM d17:1/16:0 and this disorder (Li et al. 2024). Risk genes for the development of sleep disorders have also been uncovered. Variants in the gene encoding for the lysosomal enzyme glucocerebrosidase, GBA, were widely shown to be common risk factors for Parkinson's disease and dementia with Lewy bodies (Gan‐Or et al. 2015; Sidransky et al. 2009; Nalls et al. 2013). Patients with PD who carry *GBA* variants possess stronger nonmotor symptoms, including cognitive impairment and REM sleep behavior disorder (Gan‐Or et al. 2018). However, *GBA* variants in healthy people also increase the risk of iRBD and the rate of conversion to neurodegeneration (Krohn et al. 2020). Altogether, mutations in SL metabolism genes might be considered as risk factors for the development of several neuropsychiatric disorders. Considering the strong heritability of well‐being (Vries et al. 2022; Nes and Røysamb 2015; Van de Weijer et al. 2020; Bartels 2015), it might be proposed that genetic modifications of the SL genes could contribute to the genetic determination of well‐being. In turn, the SL system might include certain risk gene variants associated with deviations of well‐being resulting in mental and psychiatric disorders. Those can further be used for prognostic purposes.

Another big area of research is focused on the potential prognostic role of SL content in biological fluids and tissues of patients for their well‐being as well as disturbances in well‐being. Here we will emphasize the potential of SL biomarkers to predict future health or well‐being outcomes rather than merely reflecting existing pathology.

Biomarkers serve diverse roles in clinical science, including diagnostic, monitoring, predictive, and prognostic applications. It is important to distinguish predictive from prognostic biomarkers: predictive biomarkers estimate the likelihood of response to a specific therapy, whereas prognostic biomarkers, the focus here, forecast the future course of a disease or health outcome independent of treatment. They provide insight into likely trajectories of disease progression, relapse risk, functional recovery, or resilience to future stressors, distinct from diagnostic biomarkers that indicate current disease presence (Biomarkers Definitions Working Group 2001; Ehrenberg et al. 2020). Establishing prognostic utility typically requires longitudinal studies tracking both biomarker levels and clinical outcomes, assessed with metrics such as sensitivity, specificity, AUC‐ROC (Area Under the Receiver Operating Characteristic Curve) for dichotomous outcomes, and hazard ratios for time‐to‐event outcomes (Cook 2007).

The central biological roles of SLs provide strong rationale for their prognostic potential. These lipids are integral to cellular structure and signaling, modulating neuroinflammation by influencing microglial activation and cytokine production (Lee, Jin, and Bae 2020), as well as neurogenesis, neuronal survival, and neuroplasticity (Echten‐Deckert 2023). Their turnover is relatively slow, suggesting that SL profiles may reflect stable, long‐term changes in neural architecture and function, making them suitable for predicting future outcomes. Chronic demyelination or persistent neuroinflammation, which can result in cognitive or mood impairments, may be indicated by specific SL signatures. Systemically, SLs regulate immune function, metabolism, and cell survival. For example, ceramide accumulation is linked to chronic stress, insulin resistance, and cellular senescence, contributing to accelerated aging and vulnerability to neurodegeneration and mood disorders (Sokolowska and Blachnio‐Zabielska 2019; Green et al. 2021). Conversely, S1P often acts as a pro‐survival, anti‐inflammatory mediator, enhancing cellular resilience and tissue repair (Echten‐Deckert 2023). Stable alterations in these pathways may therefore indicate individual vulnerability or resilience to future physiological and psychological stressors, underscoring their potential to predict long‐term well‐being.

The implementation of SLs as prognostic biomarkers depends on robust analytical approaches. SLs can be measured in plasma, serum, CSF, dried blood spots (DBS), or exosomes, each with unique advantages and limitations, and intracellular localization may also provide relevant information (Zhang et al. 2019; Turpin‐Nolan and Brüning 2020). LC–MS/MS remains the gold standard for specific species quantification, while high‐resolution mass spectrometry allows broader, untargeted profiling (Felippe et al. 2025). For prognostic purposes, biomarkers must be stable over time with minimal intra‐individual variability. Diurnal and nutritional effects should be controlled using standardized sample collection protocols (Begum et al. 2016; Voß et al. 2025). Once quantified, sophisticated statistical analyses are required to extract prognostic information from complex datasets, with ROC curves, Cox proportional hazards models, and machine learning approaches increasingly applied to integrate multiple SL species and clinical variables for enhanced predictive accuracy.

Emerging evidence supports the prognostic potential of SLs in various contexts. In neurodegenerative disease, CSF and plasma ceramide and SM profiles predict cognitive decline and conversion from mild cognitive impairment to Alzheimer's disease (Mielke, Bandaru, et al. 2010; Mielke et al. 2011, 2017). Sex‐specific associations have been observed: among men, higher ceramide and SM levels were linked to increased Alzheimer's disease risk, whereas women in the highest SM tertile had reduced risk, particularly APOE ɛ4 carriers. In Parkinson's disease, specific SL species or ratios show some associations with disease progression, but findings are inconsistent across cohorts (Couto et al. 2024). In coronary artery disease, reduced plasma ceramide C18:0 levels correlated with improvements in verbal memory, visuospatial memory, processing speed, and global cognition over 6 months of cardiac rehabilitation, suggesting its utility as a biomarker of cognitive response to exercise (Saleem et al. 2017).

SLs may also have prognostic relevance in mental health. Alterations in specific SL species have been observed in individuals at high risk for psychosis, potentially predicting conversion (van Kruining et al. 2020). Lower SM and glycerophospholipid levels, along with higher lysophospholipid levels, were linked to both prevalent and incident depression, and changes in multiple lipid classes, including SMs, glycerophospholipids, acylcarnitines, fatty acids, and triacylglycerols, correlated with shifts in depressive symptoms and psychosomatic traits. Distinct lipid network patterns were associated with depression risk (Miao et al. 2023). In stress‐related disorders, plasma SL profiles may predict vulnerability or resilience, with lower S1P or higher ceramide‐to‐S1P ratios potentially indicating worse long‐term outcomes.

Despite their promise, several challenges must be addressed to integrate SLs as prognostic biomarkers in clinical practice. Distinguishing whether SL alterations drive outcomes or reflect underlying pathology remains difficult, though stable markers of dysregulation can still hold prognostic value (Biomarkers Definitions Working Group 2001). SL profiles vary with age, sex, ethnicity, and comorbidities, necessitating validation in diverse populations. Moreover, lifestyle factors, medication use, and acute stress influence SL metabolism, requiring careful control in longitudinal studies. Finally, the lack of standardized analytical methods, quality control, and reference ranges limits comparability and translation into clinical practice.

Future directions in the field include integrating sphingolipidomics with genomics, proteomics, transcriptomics, and neuroimaging to improve predictive accuracy. Large, multi‐year prospective cohorts linking comprehensive lipidomics data to multi‐domain well‐being outcomes are critical for robust validation. Translating these findings into clinical practice will require the development of accessible, cost‐effective, and standardized SL panels for preventive screening. Ultimately, prognostic SL profiles could guide personalized interventions, including lipid‐modulating diets, targeted pharmaceuticals, or lifestyle strategies, to mitigate future adverse outcomes.

Owing to their fundamental roles in cellular and systemic biology, SLs hold considerable promise as prognostic markers for long‐term mental and physical well‐being. While early studies demonstrate utility in neurodegenerative, cardiovascular, and metabolic contexts, their potential for predicting psychiatric outcomes and broader well‐being trajectories is increasingly recognized. Rigorous validation, standardized analytical approaches, and multi‐omics integration will be essential to translate these findings into personalized preventive care, enhancing the prediction and promotion of well‐being across the lifespan.

## 6 Conclusions

Emotional well‐being is a complex, multifaceted construct that not only underlies a person's subjective quality of life but also significantly influences their mental and physical health. It comprises various dimensions, such as funding positive meaning in life events, sustaining supportive social interactions, engaging in heathy reward‐seeking behaviors, all of which manifest in everyday behavior. Among the physiological and metabolic underpinnings shaping these behaviors, SL homeostasis has now been encompassed as a potential modulator of several components of emotional well‐being. Due to their fundamental roles in the brain and peripheral organs, SLs, particularly ceramides and gangliosides, contribute to neuroplasticity, stress resilience, cognitive performance, reward‐directed behavior, and positive social engagement. In turn, dysfunctional SL metabolism could result in mental disorders. Therefore, the SL rheostat is suggested as a potential new mechanism of the key behavioral manifestations of emotional well‐being, which might determine life quality and physical and mental endurance. They emerge as not only mechanistic mediators of emotional well‐being, but also as potential biomarkers for psychological state and mental physical health as well as targets for novel interventions.

## Author Contributions

**L. S. Kalinichenko:** conceptualization, writing – original draft, writing – review and editing. **I. Zoicas:** writing – original draft, writing – review and editing. **C. Mühle:** writing – original draft, writing – review and editing. **J. Kornhuber:** writing – review and editing. **C. P. Müller:** writing – review and editing.

## Conflicts of Interest

The authors declare no conflicts of interest.

## Data Availability

The authors have nothing to report.
